# Higher Body Fat in Children and Adolescents With Type 1 Diabetes–A Systematic Review and Meta-Analysis

**DOI:** 10.3389/fped.2022.911061

**Published:** 2022-06-24

**Authors:** Yuwen Zheng, Mahdi Rostami Haji Abadi, Jonathan Gough, James J. D. Johnston, Munier Nour, Saija Kontulainen

**Affiliations:** ^1^College of Kinesiology, University of Saskatchewan, Saskatoon, SK, Canada; ^2^College of Engineering, University of Saskatchewan, Saskatoon, SK, Canada; ^3^College of Medicine, University of Saskatchewan, Saskatoon, SK, Canada

**Keywords:** type 1 diabetes (T1D), children, adolescents, body composition, body fat, lean mass, dual energy x-ray absorptimetry

## Abstract

**Aims:**

Higher prevalence of overweight and obesity in children and adolescents with type 1 diabetes (T1D) suggests alterations are required in body composition. However, differences in body composition between children with T1D and typically developing children (TDC) have not been synthesized using meta-analysis. Therefore, we conducted a systematic review and meta-analysis to compare body composition between children with T1D and TDC, and to explore the role of disease and non-disease related factors in potential body composition differences.

**Methods:**

Studies were performed comparing dual-energy x-ray absorptiometry-acquired total body fat and lean mass, absolute (kg) and relative (%) values, between children with T1D and TDC. We reported mean differences with 95% confidence intervals (CI) from meta-analysis and relative between-group %-differences. We used meta-regression to explore the role of sex, age, height, body mass, body mass index, Hemoglobin A1c, age of onset, disease duration, and insulin dosage in the potential body composition differences between children with T1D and TDC, and subgroup analysis to explore the role of geographic regions (*p* < 0.05).

**Results:**

We included 24 studies (1,017 children with T1D, 1,045 TDC) in the meta-analysis. Children with T1D had 1.2 kg more fat mass (kg) (95%CI 0.3 to 2.1; %-difference = 9.3%), 2.3% higher body fat % (0.3–4.4; 9.0%), but not in lean mass outcomes. Age of onset (β = −2.3, −3.5 to −1.0) and insulin dosage (18.0, 3.5–32.6) were negatively and positively associated with body fat % mean difference, respectively. Subgroup analysis suggested differences among geographic regions in body fat % (*p* < 0.05), with greater differences in body fat % from Europe and the Middle East.

**Conclusion:**

This meta-analysis indicated 9% higher body fat in children with T1D. Earlier diabetes onset and higher daily insulin dosage were associated with body fat % difference between children with T1D and TDC. Children with T1D from Europe and the Middle East may be more likely to have higher body fat %. More attention in diabetes research and care toward body composition in children with T1D is needed to prevent the early development of higher body fat, and to minimize the cardiovascular disease risk and skeletal deficits associated with higher body fat.

## Introduction

The prevalence of being overweight and obese is rising globally, more significantly among those with type 1 diabetes (T1D), which was determined primarily by body mass index (BMI) percentiles (1, 2). Intensive insulin therapy may be one underlying factor in the higher prevalence of children with T1D being overweight (3, 4), and the increased body mass after receiving intensive insulin therapy may be primarily due to fat accumulation (4). However, BMI cannot directly indicate fat or lean tissue inside the human body (5) and is therefore not capable of answering whether the higher prevalence of being overweight and obese in children with T1D is related to higher body fat.

A recent systematic review summarized the findings of body composition in children with T1D (6). It emphasized the concern of fat accumulation in relation to cardiovascular risk factors, including higher blood pressure (7) and blood lipids (8), in this population (6). A higher level of body fat is also a concern during growth and development (such as lower bone mass in children) (9). However, there is a lack of meta-analysis assessing body fat and lean tissue mass in children with T1D and TDC, which would help determine the direction and magnitude of potential alternations in body composition, especially in body fat, in children with T1D. In addition, no previous meta-analysis or meta-regression explored the role of potential factors, including disease-related factors, such as Hemoglobin A1c (HbA1c), in body fat in children with T1D. These findings could help guide future interventions or therapies aiming to prevent accumulating higher levels of body fat in children with T1D.

Assessments of body composition include radiographic imaging (e.g., dual-energy x-ray absorptiometry, DXA) and non-imaging based methods (e.g., bioelectric impedance, skinfold thickness) (6). DXA is considered the “gold standard” technique for total body composition assessment as it offers high accuracy and low precision errors (10, 11), while the reliability of non-imaging body composition assessment methods remains questionable in children (12, 13). Therefore, our study objectives were to perform a systematic review and meta-analysis to compare DXA-acquired body composition between children with T1D and TDC and to explore the role of disease and non-disease related factors in the potential body composition difference.

## Methods

This systematic review and meta-analysis followed the Preferred Reporting Items for Systematic Reviews and Meta-Analyses (PRISMA) 2020 guidelines (14).

### Data Sources and Search Strategy

A comprehensive literature search focusing on body composition in children and adolescents with T1D was performed using MEDLINE (1946 to June 20, 2021), EMBASE (1947 to June 20, 2021), SPORTDiscus (1937 to June 20, 2021), Web of Science (1900 to June 20, 2021), and Scopus (1947 to June 20, 2021) on June 21, 2021. We limited the search to English and human studies. Two reviewers (YZ, JG) independently performed the search. The search terms were selected to cover the following conceptual groups–children/adolescents, T1D, and body composition outcomes. Records identified from database searches were uploaded to EndNote (Version X9.3.3) for de-duplication. Detailed MEDLINE search strategies are included in Supplementary Table 1.

### Eligibility Criteria and Study Selection

Eligible articles were required to meet the following criteria: (1) population/exposure: including children and/or adolescents with T1D [reported mean or median age ≤ 18 yrs with at least 1-year mean or median disease duration (6)] and without other pre-existing health conditions which may be associated with body composition measures [e.g., hypertension (7)]; (2) study design: cross-sectional studies or baseline from intervention or longitudinal studies comparing children with T1D to TDC; (3) outcomes: DXA-derived total body fat and lean mass (kg) as well as body fat % and lean mass %; (4) full-length peer-reviewed primary research articles written in English. Detailed inclusion/exclusion criteria are included in Supplementary Table 2. The abstract review was performed independently by two reviewers (YZ, JG) via an online screening platform (15), followed by full-text screening. The disagreement was resolved by a third reviewer (MR). The reference lists from included articles were screened for additional relevant reports.

### Data Extraction Process, Data Items, and Risk of Bias Assessment

Data extraction included general and demographic information, diabetes-related background characteristics, and body composition measures, which were performed independently by two reviewers (YZ, JG). General information included authors, year, country, and study design. Demographic information included sample size, sex, age, and anthropometry measures. Disease-related background characteristics included HbA1c, disease duration, and insulin dosage. The recorded body composition outcomes included absolute values of total body fat and lean mass (kg), as well as body fat % and lean mass %.

Two reviewers (YZ, MR) assessed the quality of included studies with the modified Newcastle–Ottawa Quality Assessment Scale for cross-sectional studies (16) (Supplementary Table 3). Good, fair, or poor qualities ratings translated to low, moderate, and high risk of bias, respectively. If disagreement appeared, a third investigator (SK) resolved the conflict.

### Statistical Analysis

We included fat mass (kg), body fat %, lean mass (kg), and lean mass % in the meta-analysis. We used random-effects models to estimate the pooled mean difference and 95% confidence intervals (CI) for body composition outcomes. We also reported relative %-differences by comparing the T1D group pooled mean to the TDC group pooled mean for outcomes with significant meta-analysis results. We calculated standard deviation from standard error or 95%CI if the original publication did not report standard deviations. In addition, if the original publication reported participants' characteristics or body composition data in subgroups (e.g., males and females), we combined the subgroups and calculated the mean and standard deviation for the whole T1D or TDC groups in the analysis for three studies (17–19) based the formulae given in Cochrane Handbook (20). We performed sensitivity analysis by excluding studies with a moderate risk of bias to assess if meta-analysis results would change. Heterogeneity was evaluated by I-squared (*I*^2^). An *I*^2^ over 75% with *p* < 0.05 was considered high heterogeneity (21). We used univariate meta-regression to identify the role of these potential factors in explaining the differences in body composition outcomes between children with T1D and TDC, as well as to explore the source of heterogeneity. Potential factors included sex (female ratio, number of female/total number), age (years), body mass (kg), height (cm), body mass, BMI (kg/m^2^), HbA1c (%), age of onset (years), disease duration (years), and insulin dosage (units per kilogram per day, U/kg/day) (5, 22–24), if at least 10 studies reported these outcomes (*p* < 0.05) (25). If a study did not report the mean age of onset but reported mean age and disease duration, we estimated the mean age of onset by subtracting the mean age from the mean disease duration. If HbA1c was reported on the International Federation of Clinical Chemistry and Laboratory Medicine scale (mmol/mol), we converted HbA1C to National Glycohemoglobin Standardization Program (NGSP) scale (%) for meta-regression, since most studies reported in NGSP scale (26). In addition, we assessed the role of countries from a subgroup analysis of geographic regions (i.e., Asia, Europe, Middle East, South America, and the Pacific rim including North America and Oceania countries (*p* < 0.05) (27). Publication bias was assessed by re-displayed funnel plots after Duval and Tweedie's trim-and-fill adjustment. We performed the meta-analysis and produced forest plots using Review Manager (RevMan, Version 5.4; The Cochrane Collaboration, Oxford, UK). We also used Comprehensive Meta-Analysis Version 3 (Biostat, Englewood, NJ 2013) to perform publication bias assessment and meta-regression and to produce meta-regression graphs and re-displayed funnel plots.

## Results

### Overview of Included Studies

**Figure 1:**
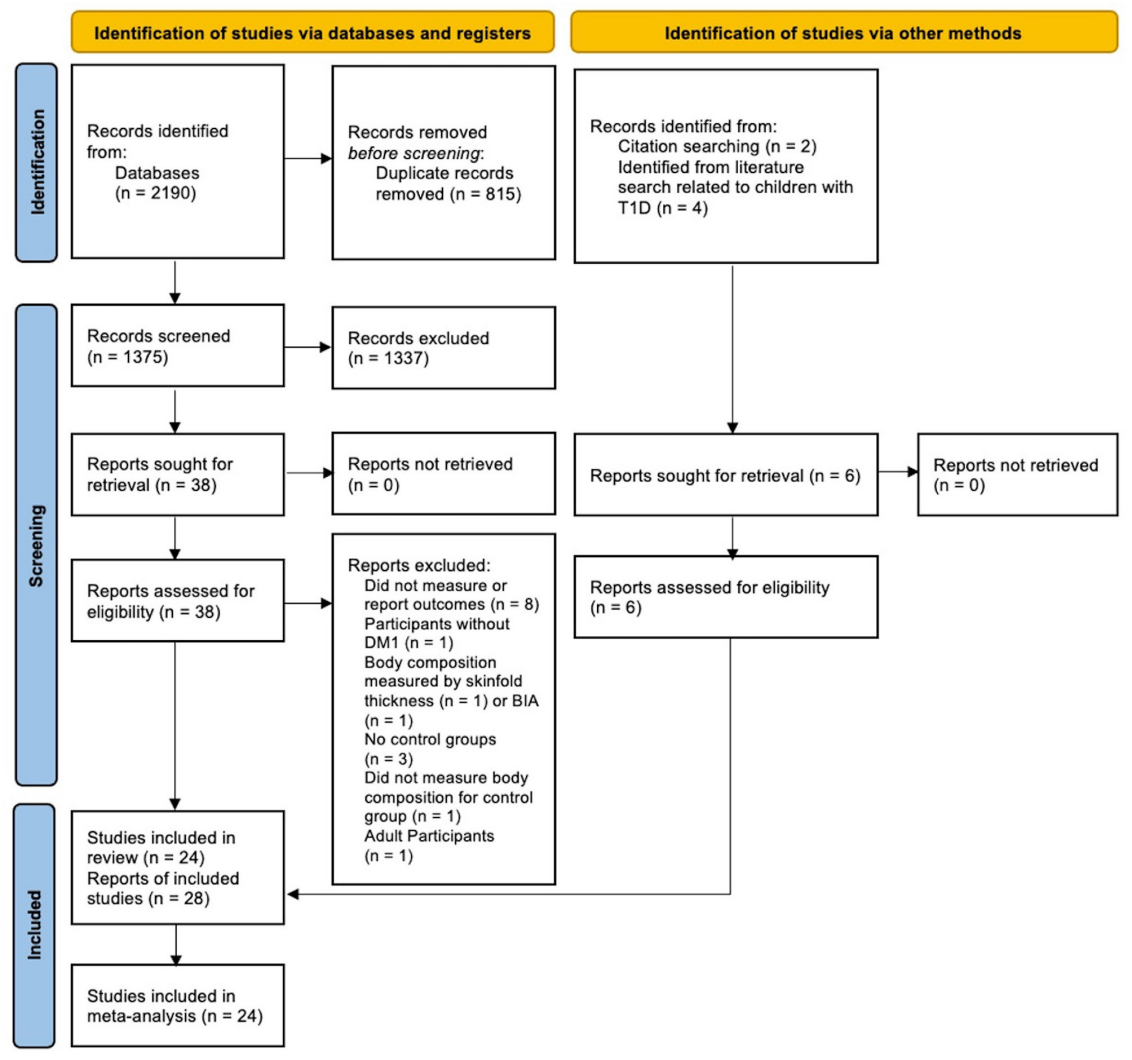
PRISMA 2020 flow diagram illustrating search and screening results.

The literature search provided 2,190 records potentially related to the area of interest; 1,375 records remained after removing duplicates. After the abstract screening, 38 reports were identified for full paper screening. We also added six additional reports to this review. Two out of the six reports were identified from screening reference lists (citation search) (28, 29). The other four reports presented body composition measures as background information (30–33). They were identified during a literature search related to children with T1D (Figure 1). We included 24 studies (28 reports) that reached all eligibility criteria in the meta-analysis (Figure 1) (14). We excluded two reports which measured body composition by bioelectric impedance or skinfold thickness, eight reports which did not measure or report the total body fat or lean composition, one study whose participants did not have T1D, one study which only included adult participants with T1D, three reports which did not have control groups, and one report which did not measure body composition for controls (Figure 1). Study participants overlapped in eight reports (13, 17, 28, 34–38). Baseline data were included in this review for follow-up or intervention studies (28, 34–36). However, baseline data reported in two reports from Gusso et al. (17, 37) was slightly different; thus, baseline data from the most recent report was included in the analysis (17). Study participants in Sarnblad et al. (13) included all study participants measured in Ingberg et al. (35), and both papers reported body fat %. As such, the body fat % reported in Sarnblad et al. (13) was included in the meta-analysis. Overall, this review included 1,017 participants with T1D and 1,045 TDC. The included studies covered publications between 2003 and 2021. The mean/median age of included study participants with T1D ranged from 10 to 17 years. The study and participants' characteristics, body composition measures, and key findings of included studies are presented in Supplementary Table 4. Twelve studies reported total body fat mass (kg), while 19 studies reported body fat. Thirteen studies reported total body lean mass (kg), while three studies reported lean mass %.

### Risk of Bias Assessment

Of the 24 included studies,19 studies had a good quality rating (low risk of bias), while five studies had a fair quality rating (moderate risk of bias) (Supplementary Table 5). The main reasons for the elevated risk of bias were (1) no control for specific background characteristics (e.g., BMI or body mass) in statistical analysis, especially when they compared the absolute value of total body fat and lean mass (kg) (7, 32, 35, 39) and (2) not using appropriate statistical method, clearly describing of statistical analysis, or report *p*-value or confidence interval (7, 35, 40).

### Meta-Analysis

**Figure 2:**
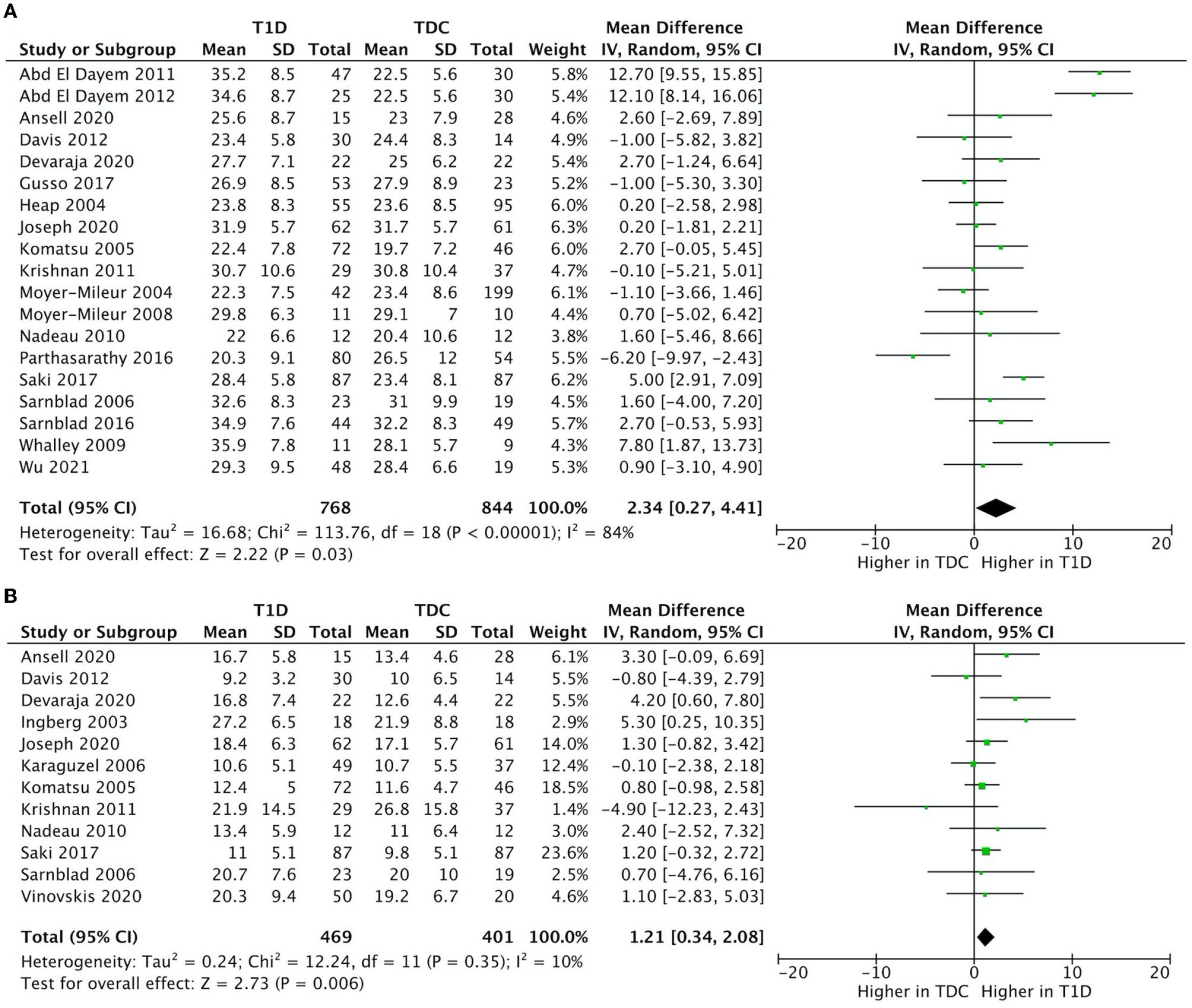
Forest plots illustrating pooled analyses of mean difference of **(A)** fat mass (kg); and **(B)** body fat % compared children with T1D to TDC, with upper and lower limits showing 95% confidence intervals.

We included all 24 studies in the meta-analysis. The results indicated that children with T1D had a higher fat mass (kg) (mean difference = 1.2; 95%CI 0.3–2.1; *n* = 12; %-difference = 9.3%) and body fat % (mean difference = 2.3; 95%CI 0.3–4.4; *n* = 19; %-difference = 9.0%) compared to TDC (Figure 2). Lean mass (kg) and lean mass % did not differ between children with T1D and TDC (Supplementary Figure 1). Sensitivity analysis (removing studies with moderate-to-high risk of bias) did not alter meta-analysis results.

There was no significant heterogeneity in fat mass (kg) (*I*^2^ = 12%, *p* = 0.330) and a moderate heterogeneity in lean mass (kg) (*I*^2^ = 46%, *p* = 0.030). Body fat % (*I*^2^ = 86%, *p* < 0.001) and lean mass % (*I*^2^ = 96%, *p* < 0.001) had high heterogeneity.

There was no evidence of publication bias in fat mass (kg), body fat %, and lean mass % (Supplementary Figure 2). There were two imputed studies in lean mass (kg) meta-analysis shown after Duvel and Tweedie's trim-and-fill adjustment, but the significance of the meta-analysis did not change after adjustment (Supplementary Figure 2).

### Meta-Regression and Subgroup Analysis

**Figure 3:**
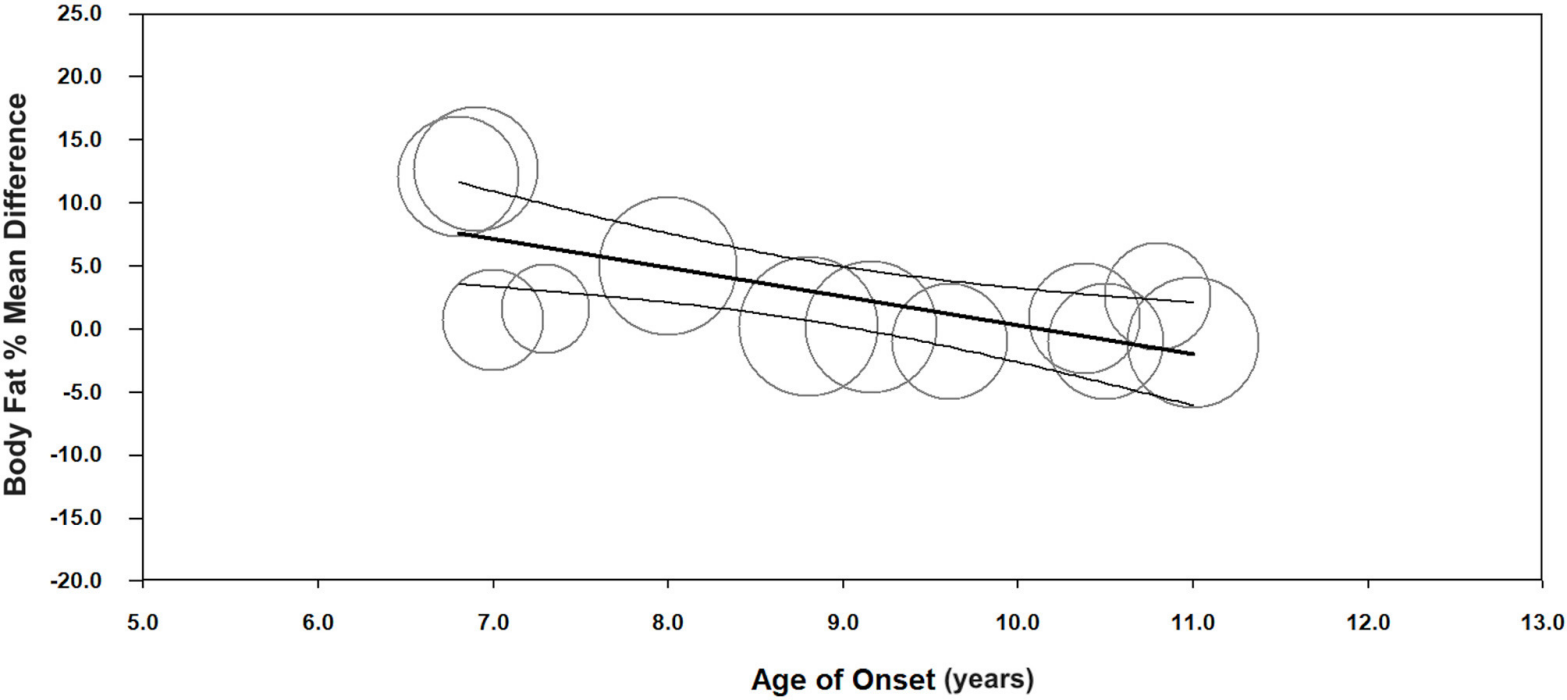
Meta-regression graph illustrating the association between age of onset (years) and body fat % mean difference, with 95% confidence intervals (CI) of body fat % compared children with T1D to TDC. The size of the circles represents the weight given to each individual study in the meta-analysis. Larger circles indicate a higher weighting.

**Figure 4:**
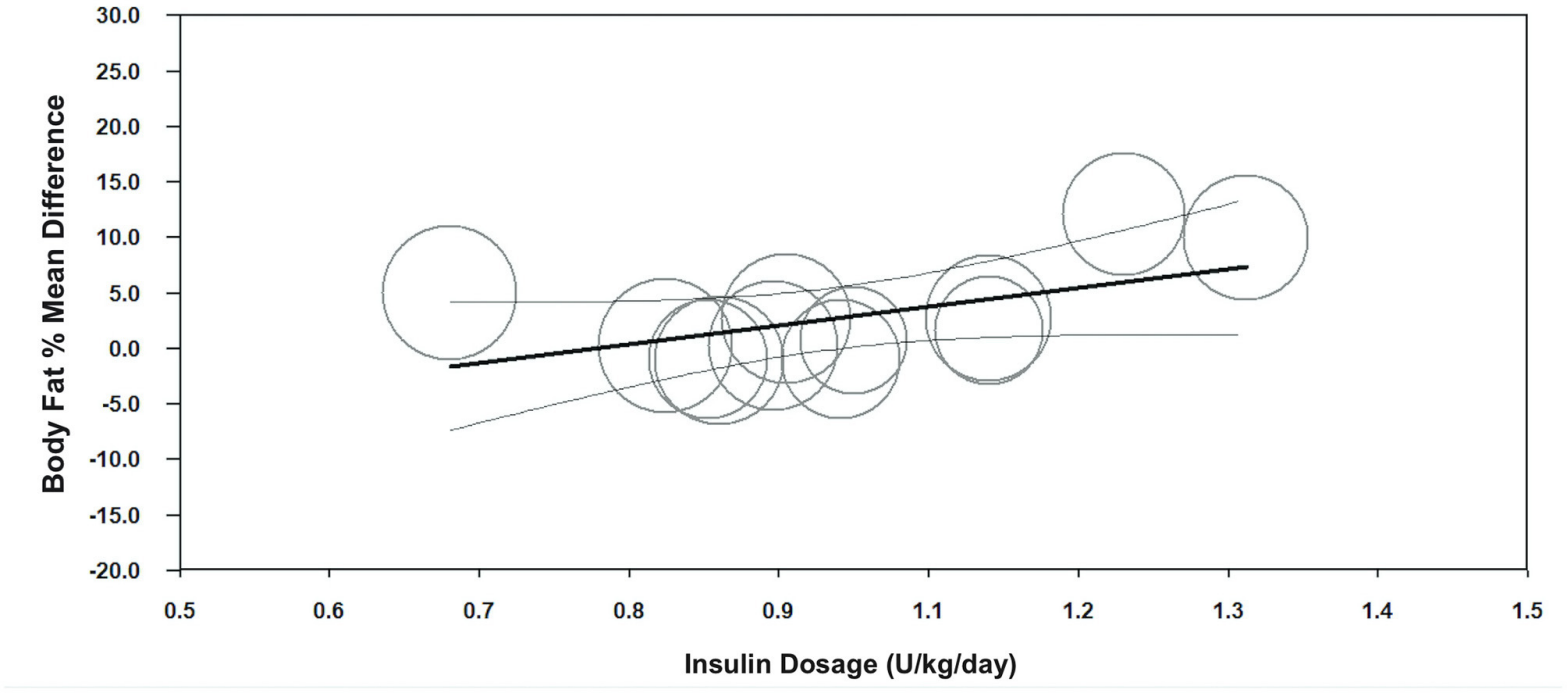
Meta-regression graph illustrating the association between insulin dosage (U/kg/day) and body fat % mean difference, with 95% confidence intervals (CI) of body fat % compared children with T1D to TDC. The size of the circles represents the weight given to each individual study in the meta-analysis. Larger circles indicate a higher weighting.

Meta-regression indicated a negative association between age of onset and body fat % (unstandardized β = −2.3, 95%CI −3.5– −1.0; *p* < 0.001, *n* = 12) (Figure 3, Supplementary Table 6), and a positive association between insulin dosage (U/kg/day) and body fat % mean difference between children with T1D and TDC (unstandardized β = 18.1, 95%CI 3.5–32.6; *p* = 0.015, *n* = 13) (Figure 4, Supplementary Table 6). Age of onset and insulin dosage explained 63 and 14% of the variance in body fat % mean difference, respectively. Female ratio, age, height, body mass, BMI, HbA1c, and disease duration did not explain variance in body fat % mean difference (*p* > 0.05) (Supplementary Table 6).

**Figure 5:**
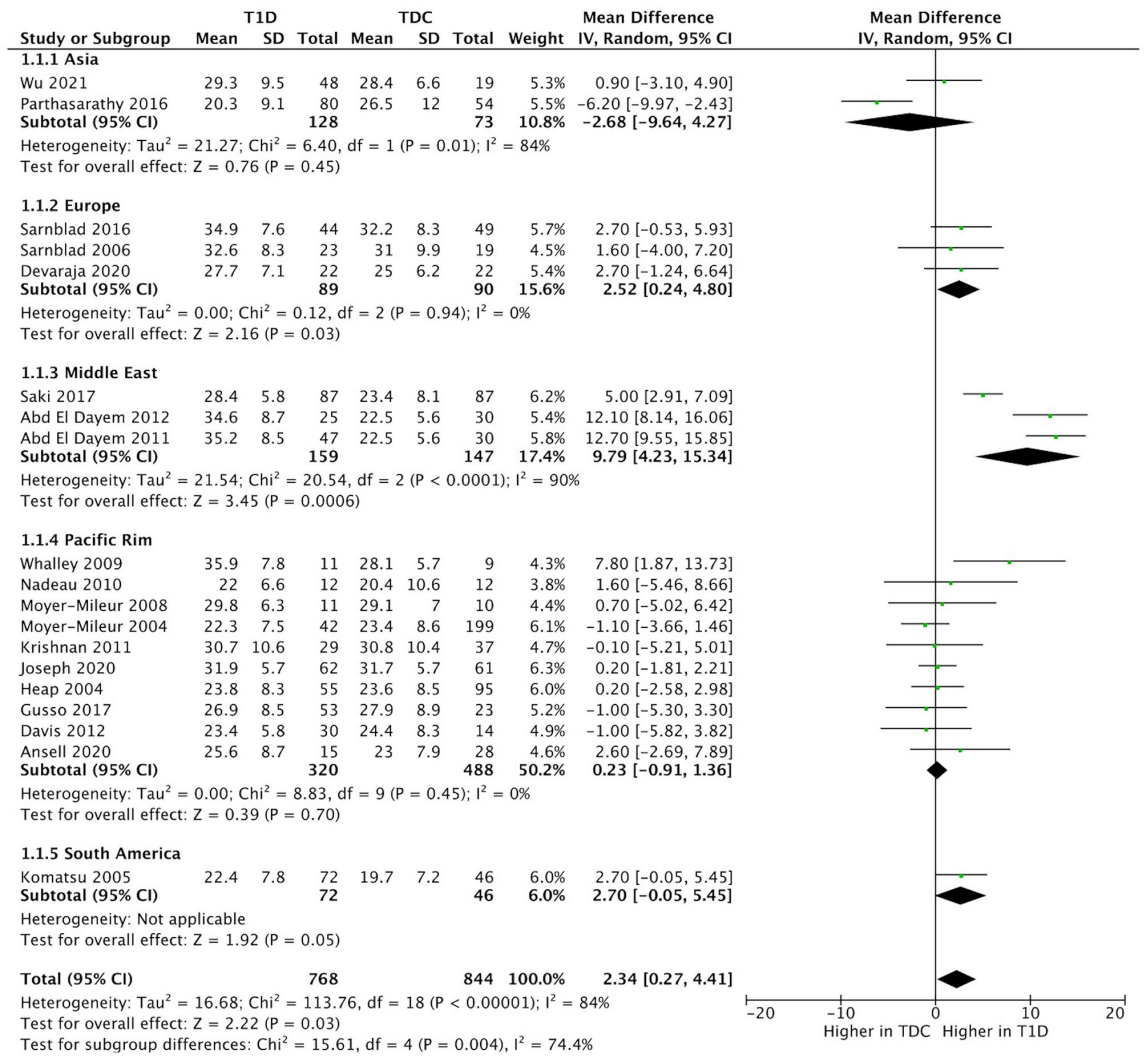
Forest plot of subgroup analysis illustrating the pooled means of body fat % across different geographic regions.

Body fat % difference varied across geographic regions (*p* < 0.001) (Figure 5). We included two studies from Asia (China, India), three studies from Europe (Sweden, UK), 10 studies from Pacific Rim (US, New Zealand), and one study from South America (Brazil). Subgroup analysis suggested a higher body fat % difference in the European (mean difference = 2.52, 95%CI 0.2–4.8, %-difference = 8.1%) and Middle East countries (mean difference = 9.8, 95%CI 4.2–15.3, %-difference = 42.8%), *vs*. similar body fat % in Asia, Pacific Rim, and South American countries (Figure 5).

## Discussion

This meta-analysis indicated a 9% higher body fat in children and adolescents with T1D compared to their typically developing peers. In absolute terms, children with T1D had an average of 1.2 kg more total body fat mass and 2.3% higher body fat %. Meta-regression results indicated that a larger daily insulin dosage (U/kg/day) was associated with a larger body fat % difference between children with T1D and TDC, but not sex, age, height, body mass, BMI, HbA1c, or disease duration.

Higher body fat % has been associated with multiple health concerns in children with T1D (6, 41, 42). A recent systematic review indicated that higher body fat % could relate to elevated blood pressure, dyslipidemia, and serum cholesterol (6). In addition, Ling et al. (41) suggested that higher body fat % was associated with slower muscle re-oxygenation after physical activity in children with T1D, which was associated with developing insulin resistance. Szadkowska et al. (42) described a negative correlation between body fat % and insulin sensitivity in children and adolescents with T1D, potentially linking to higher cardiovascular disease risk. Higher body fat may also relate to the skeletal deficits in children with T1D (9). Children with T1D have lower bone mass (i.e., mineral content and areal bone mineral density) along with altered trabecular density and micro-architecture (e.g., trabecular thickness) according to meta-analyses (43–45). Recent evidence suggested that body fat % was negatively associated with total body bone mineral content and areal bone mineral density in children (9). There are several potential mechanisms behind this negative relationship. For example, more fat accumulation may favor the adipogenic process, reducing bone formation as both osteoblasts and adipocytes are generated from bone marrow mesenchymal stem cells (46). Also, adipose tissue can secrete proinflammatory cytokines, promoting osteoclast activity and bone resorption (9). Therefore, potential methods to prevent elevating body fat levels, such as enhancing insulin injection strategies to lower the insulin dosage, are warranted for further exploration in children living with T1D. Such actions may help lower the potential for risk to develop cardiovascular disease and skeletal deficits associated with a high level of body fat.

There are several potential explanations for higher body fat % in children with T1D. Early diabetes onset and a higher dose of exogenous insulin treatment are possible explanations. A study suggested the earlier onset of T1D is associated with higher body mass and fat at 6-week and 1-years post-diagnosis, which may be related to the earlier development of insulin resistance (47). In addition to the age of onset, insulin may inhibit the catabolic process of lipid and promote lipogenesis (4), which induces higher weight gain and fat accumulation (48–50). Furthermore, higher insulin dosage may lead to higher leptin which contributes to body fat accumulation in girls (51). Meta-regression results also support the role of insulin dosage in the difference in body fat %, which implies that a larger daily dosage of insulin (U/kg/day) may contribute to higher body fat % in children with T1D. On the other hand, previous longitudinal evidence suggested that higher HbA1c is associated with lower BMI but higher insulin dosage in children and adolescents with T1D (23). However, HbA1c may not relate to the difference in body fat % between children with T1D and TDC, as our meta-regression suggested HbA1c in children with T1D did not contribute to the difference in body fat % between children with T1D and TDC (Supplementary Table 6). Other than disease-related factors, geographic location may play a role, as studies from Europe and the Middle East reported a greater difference in body fat % between children with T1D and their healthy peers. This finding suggests that environmental factors, including economic background, and/or ethnicity may play a role in body fat % differences. It has to be noted that our sample size for the subgroup analysis was small, and we were unable to assess related factors, such as the role of ethnicity because it was not systematically reported in the studies included in the meta-analysis. Future studies are needed to explore the role of geographic location, including socioeconomic, ethnic, cultural, and lifestyle factors in the body composition differences between children with T1D and TDC.

In terms of lifestyle factors, higher body fat % in children with T1D may relate to an unhealthy diet and lower physical activity (6). According to a large cohort study on diet patterns in youth with T1D, over 90% of participants exceeded the recommendation for saturated fat intake (52). Children with T1D also had higher than recommended intakes of sugar and fat but intakes did not differ from TDC (53). Similar energy intake but more sedentary time and lower physical activity in children with T1D, when compared to TDC, may contribute to the differences in body composition (54). Lower physical activity in children with T1D may relate to reported barriers, mainly the fear of post-exercise hypoglycemia (55). Unfortunately, only a limited number of studies reported physical activity measures for both T1D and TDC groups (19, 28, 29, 31, 32, 39, 56–58). Also, these studies measured physical activity using a variety of ways, including different questionnaires or accelerometers. Therefore, we were not able to explore the role of physical activity in the higher body fat % in children with T1D. Of note, two studies reported less physical activity (counts per minute) in children with T1D, both of which used objective accelerometer measures (28, 31). None of the self-reported questionnaires assessed physical activity outcomes, including the metabolic cost of activity, the number of hours of physical activity per week, or physical activity score, which differed between children with T1D and TDC (19, 28, 29, 56–58). More studies linking objectively measured physical activity outcomes, especially clinically relevant outcomes like minutes of moderate-to-vigorous physical activity, to the body composition would enhance our understanding of the potential role of physical activity in higher body fat in children with T1D.

Meta-analyses suggested lean mass, both absolute- and %-based, did not differ between children with T1D and TDC. This implies that children with T1D may not have alterations in skeletal muscle mass, as lean mass measured by DXA is predominantly skeletal muscle mass and highly correlated to muscle mass measured by computed tomography (CT) or magnetic resonance imaging (in adults) (11, 59). Another surrogate of skeletal muscle mass, muscle cross-sectional area was normal-to-higher in adolescents with T1D in the forearm and a lower leg compared to reference or control groups, assessed by peripheral quantitative CT (19, 60, 61). Although smaller muscle area was observed in pre-pubertal or early-pubertal children with T1D, muscle area was comparable to the reference population in adolescents with T1D (60, 62).

To the best of our knowledge, this study is the first meta-analysis comparing DXA-derived body composition outcomes in children with T1D to TDC. DXA imaging is considered an accurate and precise tool to assess body composition in children (10, 11). It is worth noting that the relative %-differences between T1D and TDC (9%) exceeded the least significant changes reported for pediatric DXA measures of fat mass (kg) (6.4%) and body fat % (6.3%) (63). This supported the premise that the differences in fat mass (kg) and body fat % observed from the meta-analysis exceeded the DXA measurement error. Other commonly used technology, like bioelectrical impedance, may not be able to assess fat and lean mass in children reliably. This is because the variation in the hydration level may influence lean mass and body fat % measures, especially in individuals with T1D due to the lower level of extracellular water and exchangeable potassium (12). Additionally, manual methods like skinfold thickness had poor accuracy in children (13). However, the meta-analysis results should be interpreted with the following considerations. There was high heterogeneity in body fat % in the meta-analysis. Although meta-regression suggested that age of onset and insulin dosage were sources of heterogeneity in the body fat % meta-analysis, unexplained heterogeneity remained.

In conclusion, children and adolescents with T1D have an average of 9% more fat in the total body than compared with TDC. Earlier diabetes onset and higher insulin dosage (U/kg/day) were associated with a larger body fat % difference between children with T1D and TDC. Body fat % difference between children with T1D and TDC may also vary across geographic regions. There was no difference in total body lean mass (kg or %) between children with T1D and TDC. Body composition in children with T1D requires more attention in diabetes research and care to prevent the early development of higher body fat and to minimize the cardiovascular disease risk and skeletal deficits associated with the higher body fat.

## Data Availability Statement

The original contributions presented in the study are included in the article/Supplementary Material, further inquiries can be directed to the corresponding author.

## Author Contributions

SK and YZ: conceptualization. YZ: methodology and formal analysis. YZ and JG: data collection. YZ, MR, JJ, MN, and SK: interpretation. YZ, MR, JG, JJ, MN, and SK: manuscript preparation. All authors contributed to the critical revision and approval of the final manuscript.

## Funding

This study was supported by the following trainee grants: YZ [PhD scholarship from Saskatchewan Center for Patient-Oriented Research (SCPOR)], MR (Dean's PhD scholarship from University of Saskatchewan, USask and Mitacs Canada scholarship), and JG (Natural Sciences and Engineering Research Council undergraduate student research award, NSERC USRA). The matching funds for the trainee grants were from the following grants: Collaborative Innovation Development Grant (Saskatchewan Health Research Foundation, SHRF), the Patient-Oriented Research Leadership Grant (SHRF and SCPOR) and NSERC Discover Grant (Natural Sciences and Engineering Research Council, Canada), and Diabetes Canada.

## Conflict of Interest

The authors declare that the research was conducted in the absence of any commercial or financial relationships that could be construed as a potential conflict of interest.

## Publisher's Note

All claims expressed in this article are solely those of the authors and do not necessarily represent those of their affiliated organizations, or those of the publisher, the editors and the reviewers. Any product that may be evaluated in this article, or claim that may be made by its manufacturer, is not guaranteed or endorsed by the publisher.
