# Supplementary material for: Higher Body Fat in Children and Adolescents With Type 1 Diabetes–A Systematic Review and Meta-Analysis
Source: Front Pediatr. 2022 Jun 24;10:911061. doi: 10.3389/fped.2022.911061 (PMC9263393; doi:10.3389/fped.2022.911061)
Supplement: Supplementary file 1 [file Data_Sheet_1.docx]

Supplementary Material

**Supplementary Table 1.** MEDLINE search strategy

| **Conceptual groups** | **Search Terms** |
| --- | --- |
| **Children/adolescents (population)** | 1. adolescent/ or child/ or child, preschool/  2. (child or children).ti,ab,kf.  3. (girl or girls). ti,ab,kf.  4. (boy or boys).ti,ab,kf.  5. (adolesc* or preadolsc* or pre-adolesc*).ti,ab,kf.  6. (teen* or preteen* pre-teen*).ti,ab,kf.  7. (pubert* or prepubert* or pre-pubert*).ti,ab,kf.  8. (pubesc* or prepubesc* or pre-pubesc*).ti,ab,kf.  9. (youth or youths).ti,ab,kf.  10. (juvenile or juveniles).ti,ab,kf.  11. (p?ediatric or p?ediatrics).ti,ab,kf.  12. 1 or 2 or 3 or 4 or 5 or 6 or 7 or 8 or 9 or 10 or 11 |
| **Type 1 diabetes (exposure)** | 1. Diabetes Mellitus, Type 1/ 2. (IDDM or T1DM or T1D or DM1).ti,ab,kf. 3. ((“insulin depend*” or “insulin-depend*” or “insulindepend*).ti,ab,kf. 4. ((diabet* or dm) adj3 (“type one” or “type 1” or “type I”)).ti,ab,kf. 5. ((juvenile or auto-immun* or autoimmun* or “sudden onset”) adj2 (diabet* or DM)).ti,ab,kf. 6. 13 or 14 or 15 or 16 or 17 7. exp Diabetes Insipidus/ 8. diabet* insipidus.ti,ab,kf. 9. 19 or 20 10. 18 not 21 |
| **Bone (outcomes)** | 23. exp Body Composition/  24. (body adj3 (composition or distribution)).ti,ab,kf.   1. ((fat or adipos*) adj3 (volume or composition or distribution or mass or index or kg or kilogram or kilograms or total or total-body or whole or whole-body)).ti,ab,kf. 2. ((fat or adipos*) adj3 (percentage or percent or “per cent” or %)).ti,ab,kf. 3. ((musc* or lean or fat-free or “fat free”) adj3 (volume or composition or distribution or mass or index or kg or kilogram or kilograms or total or total-body or whole or whole-body)).ti,ab,kf. 4. ((musc* or lean or fat-free or “fat free”) adj3 (percentage or percent or “per cent” or %)).ti,ab,kf. 5. ((android or gynoid or visceral or appendicular or abdominal or intra-abdominal) adj3 (fat or lean or muscle or mass or adipos*)).ti,ab,kf. 6. 23 or 24 or 25 or 26 or 27 or 28 or 29 |
| **Combined** | 1. 12 and 22 and 30 2. Limit: English |

Database: Ovid MEDLINE(R) and Epub Ahead of Print, In-Process & Other Non-Indexed Citations, Daily and Versions(R)

**Supplementary Table 2.** Inclusion/Exclusion criteria for study selection and excluded reports

| Considered Items | Inclusion | Exclusion | Excluded Reports |
| --- | --- | --- | --- |
| Study Design | Cross-sectional studies or baseline from intervention or longitudinal studies compared with TDC | Follow-up or post-intervention data from intervention group in studies with interventions  Studies without compared to typically developing controls | No control groups (1–3) |
| Study population | Children and adolescents with diabetes mellitus, type 1 (mean or median age $\leq$18yrs) with (1) at least one year mean or median disease duration, and (2) no other conditions or medication potentially influencing body composition (e.g., hypertension) | Adults with diabetes mellitus, type 1 (mean or median age $>$18yrs)  Children and adolescents with diabetes mellitus, type 1 with less than one year mean or median disease duration  Children and adolescents with diabetes mellitus, type 1 with other conditions or medication potentially influencing body composition (e.g., hypertension) | Participants without T1D (4)  Adult participants with T1D (5) |
| Outcomes | At least one of body fat %, lean mass%, total body fat and lean mass measured by DXA | Outcomes measured by other technology (e.g., bioelectrical impedance, skinfold thickness)  No outcomes reported | Measured by bioelectrical impedance (6), and skinfold thickness (7)  Did not measure or report total body composition outcomes (8–15)  Did not measure or report total body composition outcomes for controls (16) |
| Publication date range | Up to Jun 20, 2021 |  |  |
| Language restriction | English only | Not English |  |
| Publication type | Original study with full text and peer reviewed | Non-original studies or without full text or peer review |  |
| Other | Human only | Animal study |  |

**Supplementary Table 3.** Newcastle-Ottawa quality assessment scale (adapted for cross sectional studies) (17)

| **Categories** | **Questions** |
| --- | --- |
| **Selection**  (5 stars max) | 1. Representativeness of the sample:    1. Truly representative of the average in the target population. * (all subjects or random sampling)    2. Somewhat representative of the average in the target population. * (non-random sampling)    3. Selected group of users.    4. No description of the sampling strategy.   *Note: If participants with type 1 diabetes and were recruited from medical clinics, like diabetes clinics, option b) would be selected*   1. Sample size:    1. Justified and satisfactory. *    2. Not justified. 2. Non-respondents:    1. Comparability between respondents and non-respondents characteristics is established, and the response rate is satisfactory. *    2. The response rate is unsatisfactory, or the comparability between respondents and non-respondents is unsatisfactory.    3. No description of the response rate or the characteristics of the responders and the non-responders. 3. Ascertainment of the exposure (risk factor):    1. Validated measurement tool. **    2. Non-validated measurement tool, but the tool is available or described.*    3. No description of the measurement tool.   *Note: if diabetes were diagnosed with type 1 diabetes, option b) would be selected* |
| **Comparability** (2 stars max) | 1. The subjects in different outcome groups are comparable, based on the study design or analysis. Confounding factors are controlled.    1. The study controls for the most important factor (Body mass/BMI (18,19)). *    2. The study control for any additional factor (e.g., sex, age, maturity, height (19–21)). * |
| **Outcome**  (3 stars max) | 1. Assessment of the outcome:    1. Independent blind assessment. **    2. Record linkage. (i.e., DXA-measured body composition) **    3. Self report. *    4. No description. 2. Statistical test:    1. The statistical test used to analyze the data is clearly described and appropriate, and the measurement of the association is presented, including confidence intervals and the probability level (*p* value). *    2. The statistical test is not appropriate, not described or incomplete   *Note: if body mass or BMI differed between groups, but not adjusted in statistical*  *analysis, it will be considered inappropriate statistical test, so option b) would be*  *selected* |

**Supplementary Table 4.** Included study characteristics

| **Reference** | **Design** | **T1D**  **Sample Size (Sexes)**  **Demographics**  Mean (SD) * | **TDC**  **Sample Size (Sexes)**  **Demographics**  Mean (SD) * | **Outcomes** | **T1D**  Mean (SD) * | **TDC**  Mean (SD) * | **Key Findings** |
| --- | --- | --- | --- | --- | --- | --- | --- |
| Abd El Dayem et al. (2011) Egypt (22) | Cross-sectional | N=47 (14M,33F) Age: 13.3yrs (3.37) Height (SDS): -0.7 (1.0) Body mass (SDS): -0.04 (1.3) BMI: 22.0kg/m^2^ (8.0) Disease duration: 6.3yrs (3.0) HbA1c: 8.8% (2.1)  Insulin dosage: 1.3 U/kg/day (0.5) | N=30 (12M, 18F) Age: 11.9yrs (2.98) BMI: 21.2kg/m^2^ (7.5) HbA1c: 5.6% (1.1) | Body fat % | 35.2 (8.5) | 22.5 (5.6) | 56% higher body fat % and 17% lower lean mass % in children with T1D |
|  |  |  |  | Lean mass % | 63.2 (8.6) | 76.1 (5.6) |  |
| Abd El Dayem & Battah (2012)  Egypt (23) | Cross-sectional | N=25 (no hypertension) Age: 13.5yrs (3.1) Height (SDS): -0.7 (1.0) Body mass (SDS): -0.0 (1.3) BMI: 20.1kg/m^2^ (3.1) BMI (Z-score): 0.8 (0.8) Disease duration: 6.8yrs (2.1) HbA1c: 8.4% (2.0)  Insulin dosage: 1.2 U/kg/day (0.3) | N=30 | Body fat % | 34.6 (8.7) | 22.5 (5.6) | Higher fat mass % in children with T1D (no hypertension) |
| Ansell et al. (2020) US (24) | Cross-sectional | N=15 (9M, 6F)  Age: 17.7yrs (1.7)  Height: 170.3cm (8.3)  Body mass: 67.3kg (8.6)  BMI: 23.2kg/m^2^ (2.0)  BMI percentile: 67 (20)  HbA1c: median 8.3% [IQR 7.2-8.9]  Disease duration: 6.9yrs (4.6) | N=28 (14M, 14F)  Age: 17.6yrs (1.7)  Height: 169.5cm (9.9)  Body mass: 60.2kg (8.6)  BMI: 20.9kg/m^2^ (1.7)  BMI percentile: 44 (21)  HbA1c: median 5.2% [IQR 4.8-5.6] | Body fat % | 23.0 (7.9) | 25.6 (8.7) | No difference in body fat %, fat and lean mass between children with T1D and TDC |
|  |  |  |  | Fat mass (kg) | 16.7 (5.8) | 13.4 (4.6) |  |
|  |  |  |  | Lean mass (kg) | 46.2 (8.7) | 43.7 (8.9) |  |
| Davis et al. (2012) ^†^ US (25) | Follow-up | N=30 (18M, 12F) Age: 10.5yrs (2.9) at baseline Height (SDS): 0.0 (1.0) Body mass (SDS): 0.1 (1.1) BMI (SDS): 0.2 (1.1) HbA1c:  M: 8.8% (1.2)  F: 7.8% (10)  Disease duration: 1yr  Insulin dosage: 0.9 U/kg/day (0.2) | N=14 (8M, 6F) Age: 10.0 (2.9) at baseline Height (SDS): 0.4 (1.5) Body mass (SDS): 0.1 (1.5) BMI (SDS): -0.2 (1.3) | Body fat % | 23.4 (5.8) | 24.4 (8.3) | No difference on body fat %, total body fat and lean mass between children with T1D at 1 year after diagnosis and TDC |
|  |  |  |  | Fat mass (kg) | 9.2 (3.2) | 10.0 (6.5) |  |
|  |  |  |  | Lean mass (kg) | 30.9 (9.9) | 29.6 (9.6) |  |
| Devaraja et al. (2020) UK (26) | Cross-sectional | N=22 (9M,13F) Age: 13.8yrs (1.2) Height: 160.6cm (9.4) Body mass: 58.1kg (14.6) BMI: 22.4kg/m^2^ (4.4) Disease duration: range from 2 months to 14.5yrs HbA1c: 62.4mmol (5.4) | 22 (9M,13F) Age: 13.6yrs (1.2) Height: 159.7cm (10.2) Body mass: 49.8kg (10.2) BMI: 19.3kg/m^2^ (2.5) | Body Fat % | 27.7 (7.1) | 25.0 (6.2) | 11-34% higher total body fat and lean mass in children with T1D; no difference on body fat % between children with T1D and TDC  No difference after adjusting for height and weight between children with T1D and TDC |
|  |  |  |  | Fat mass (kg) | 16.8 (7.4) | 12.6 (4.4) |  |
|  |  |  |  | Lean mass (kg) | 41.5 (8.3) | 37.3 (7.74) |  |
| Gusso et al. (2017) New Zealand (27) | Randomized controlled trial | T1D Intervention Group:  N=38 (20M, 18F)  Age: 15.6yrs (1.3)  Body mass: 69.8kg (95% CI 66.1–73.6)  BMI: 23.5kg/m^2^ (95% CI 22.4–24.7)  HbA1c: 8.8% (95% CI 8.4–9.3)  Disease duration: 5.4yrs (3.4)  T1D Control Group:  N=15 (7M, 8F)  Age: 15.5yrs (0.9)  Body mass: 69.2kg (95% CI 63.3–75.1)  BMI: 24.6kg/m^2^ (95% CI 22.8–26.4)  HbA1c: 8.6% (95% CI 7.8–9.3)  Disease duration: 7.5yrs (4.0) | N=22 (10M, 12F) Age: 16.7yrs (1.5) Body mass: 64.6kg (95% CI 59.5–69.7) BMI: 23.0kg/m^2^ (95% CI (21.4–24.5) HbA1c: 5.2% (95% CI (4.4–5.9) | Body Fat % | T1D Intervention  26.8 (8.6) | TDC Intervention  27.9 (8.9) | No difference on body fat % across T1D intervention, control groups and non-diabetic controls at baseline |
|  |  |  |  |  | T1D Control  27.3 (8.5) |  |  |
| Heap et al. (2004) US (28) | Cross-sectional | N=55 (30M,25F) Age: M: 14.6yrs (1.7) F: 14.7yrs (1.9) Height: M: 165.4cm (13.1) F: 161.8cm (8.2) Body mass:  M: 62.4 kg (17.0) F: 61.3 kg (17.8) BMI: M: 22.6 kg/m^2^ (4.7) F: 23.2 kg/m^2^ (5.2) Disease duration:  Tanner Stage II: 3.8yrs (3.7)  Tanner Stage III: 3.7yrs (3.0)  Tanner Stage IV: 5.8yrs (4.3)  Tanner Stage V: 6.7yrs (3.6)  Average HbA1c:  Tanner Stage II: 9.4% (2.6)  Tanner Stage III: 8.0% (0.7)  Tanner Stage IV: 9.0% (1.5)  Tanner Stage V: 9.0% (1.4)  Insulin dosage:  Tanner Stage II: 0.8 U/kg/day (0.3)  Tanner Stage III: 1.0 U/kg/day (0.2)  Tanner Stage IV: 1.0 U/kg/day (0.3)  Tanner Stage V: 0.9 U/kg/day (0.3) | N=95 (42M,53F) Age:  M: 14.5yrs (1.9) F: 14.8yrs (1.5) Height:  M: 166.8cm (12.2) F: 161.0cm (8.3) Body mass: M: 58.2 kg (13.7) F: 57.8 kg (15.7) BMI: M: 20.7 kg/m^2^ (3.2) F: 22.1 kg/m^2^ (4.9) | Body fat % | 23.8 (8.3) | 23.6 (8.5) | No difference on body fat % and total body lean mass between children with T1D and TDC |
|  |  |  |  | Lean mass (kg) | 42.1 (10.4) | 41.1 (10.9) |  |
| Ingberg et al. (2003); Sweden (29) | Cross-sectional | N=18 (18F) Age: 17.3yrs (0.6) Height: 166.0cm (7.0) Body mass: 72.6kg (8.4) BMI: 26.3kg/m^2^ (2.6) Disease duration: 9.3yrs (3.2) HbA1c: 8.0% (1.1)  Insulin dosage: 1.1 U/kg/day (0.3) | N=18 (18F) Age: 17.3yrs (0.6) Height: 170cm (6) Body mass: 65.8kg (12.5) BMI: 23.6kg/m^2^ (3.8) | Body Fat % | 37.1 (5.5) | 32.1 (7.7) | 16% higher body fat % and 24% higher total body fat mass in girls with T1D |
|  |  |  |  | Fat mass (kg) | 27.2 (6.5) | 21.9 (8.8) |  |
| Joseph et al. (2020)  US (30) | Cross-sectional | N=62 (62F) Age: 13.6yrs (1.7) Height (Z-score): 0.3 (1.1) Body mass (Z-score): 0.7 (0.8) BMI (Z-score): 0.7 (0.7) Diabetes duration: 4.8yrs (3.2) HbA1c: 8.6% (1.3)  Insulin dosage: 0.9 U/kg/day (0.2) | N=61 (61F) Age: 13.6yrs (1.9) Height (Z-score): 0.4 (0.9) Body mass (Z-score): 0.4 (0.9) BMI (Z-score): 0.3 (0.8) HbA1c: 5.4% (0.3) | Body fat % | 31.9 (5.7) | 31.7 (5.7) | No difference on body fat %, total body fat and lean mass between children with T1D and TDC |
|  |  |  |  | Fat mass (kg) | 18.4 (6.3) | 17.1 (5.7) |  |
|  |  |  |  | Lean mass (kg) | 36.5 (6.1) | 34.2 (6.8) |  |
| Karaguzel et al. (2006)  Turkey (31) | Cross-sectional | N=49 (26M,23F) Age: 11.3yrs (2.8) Body mass:  M: 38.4kg (10.9) F: 37.2kg (15.2) BMI: 17.9kg/m^2^ (3.0) Disease duration: M: 4.2yrs (3.1) F: 3.9yrs (3.0) Average HbA1c: HbA1c (<8%): N=18 HbA1C (>8%): N=31  Insulin dosage: 0.9 U/kg/day (0.3) | N=37 (20M,17F) Age: 11.0yrs (3.1) Body mass:  M: 42.5 (14.2) F: 37.2 (14.8) BMI: 18.2kg/m^2^ (3.0) | Lean mass (kg) | Male  28.0 (9.5) | 28.8 (13.0) | No difference on total body fat and lean mass between children with T1D and TDC |
|  |  |  |  |  | Female  21.6 (7.4) | 22.4 (8.5) |  |
|  |  |  |  | Fat mass (kg) | Male  9.0 (3.0)  Female  12.5 (6.4) | 9.0 (4.4)  12.8 (6.0) |  |
| Komatsu et al. (2005) Brazil (32) | Cross-sectional | N=72 (38M,34F) Age: median 16yrs (range 9-20) Height: 160cm (12) Body mass: 56.0kg (13.4) BMI: 21.5kg/m^2^ (3.7) Disease duration: 4.9yrs (3.6) HbA1c: 8.1% (2.2)  Insulin dosage: 1.0 U/kg/day (0.4) | N=46 (26M,20F) Age: median 16yrs (range 10-18) Height: 166cm (10) Body mass: 58.2kg (12.7) BMI: 20.8kg/m^2^ (2.8) HbA1c: 5.2% (0.9) | Body fat % | 22.4 (7.8) | 19.7 (7.2) | No difference on body fat %, total body fat and lean mass between children with T1D and TDC |
|  |  |  |  | Fat mass (kg) | 12.4 (5.0) | 11.6 (4.7) |  |
|  |  |  |  | Lean mass (kg) | 41.2 (10.0) | 45.0 (9.5) |  |
| Krishnan et al. (2011) US (33) | Cross-sectional | N=29  Normal Weight (N=14; 9M,5F)  Age: 15.9yrs (2.3)  Height: 162.5cm (23.7)  Body mass: 59.0kg (11.3)  BMI: 20.6kg/m^2^ (2.0)  BMI percentile: 50.7% (21.6)  Disease duration: >3yrs HbA1c: 8.8% (1.3)  Overweight (N=15; 10M,5F)  Age: 16.3yrs (2.2)  Height: 171.1cm (8.7)  Body mass: 84.3kg (17.6)  BMI: 28.5kg/m^2^ (3.6)  BMI percentile: 93.1% (4.2)  Disease duration: >3yrs  HbA1c: 8.2% (1.0) | N=37  Normal Weight (N=14; 6M,8F)  Age: 16.5yrs (2.6)  Height: 167.2cm (6.9)  Body mass: 57.4kg (7.5)  BMI: 20.5kg/m^2^ (1.5)  BMI percentile: 47.3% (17.1)  HbA1c: 5.1% (0.3)  Overweight (N=23; 13M,10F)  Age: 15.6yrs (2.1)  Height: 168.3cm (9.9)  Body mass: 93.7kg (19.9)  BMI: 32.9kg/m^2^ (5.6)  BMI percentile: 96.6% (3.2)  HbA1c: 5.4% (0.2) | Body fat % | Normal Weight | | No difference on body fat %, total body fat mass between children with T1D and TDC |
|  |  |  |  |  | 24.3 (1.7) | 21.4 (1.6) |  |
|  |  |  |  |  | Overweight  36.7 (2.7) | 36.5 (1.7) |  |
|  |  |  |  | Fat mass (kg) | Normal Weight | |  |
|  |  |  |  |  | 13.4 (1.1) | 11.3 (1.1) |  |
|  |  |  |  |  | Overweight  29.9 (4.2) | 36.2 (2.6) |  |
| Maggio et al. (2010) Switzerland (34) | Cross-sectional | N=27 (13M,14F) Age: 10.5yrs (2.4) Height: 141.8cm (15.1) Body mass: 38.3kg (12.0) BMI: 18.5kg/m^2^ (12.0) Disease duration: 3.2yrs (SE 12.0) HbA1c: 7.9% (3.6)  Insulin dosage: 0.8 U/kg/day (0.2) | N=32 (16M,16F) Age: 10.5yrs (2.5) Height: 143.1cm (15.8) Body mass: 37.3kg (13.0) BMI: 17.6kg/m^2^ (SE 2.6) HsbA1c: 5.4% (3.3) | Lean mass (kg) | 28.5 (9.9) | 27.7 (9.1) | No difference on total body lean mass between children with T1D and TDC |
| Moyer-Mileur et al. (2004) US (35) | Follow-up | N=42 (26M,16F) Age: M: 14.9yrs (1.8) F: 14.1yrs (1.8) Height:  M: 166cm (12) F: 161cm (7) Body mass: M: 62.1kg (23.8) F: 53.2kg (8.5) BMI: M: 23.4kg/m^2^ (6.9) F: 20.5kg/m^2^ (3.5) Disease duration: 4.2yrs (3.1) HbA1c: 8.2% (1.2)  Insulin dosage: 0.9 U/kg/day (0.3) | N=199 (90M,109F) Age: M: 15.0yrs (2.1) F: 15.1yrs (1.9) Height:  M: 168cm (12) F: 162cm (8) Body mass:  M: 59.8kg (17.1) F: 58.2kg (15.4) BMI: M: 21.0kg/m^2^ (4.6) F: 22.1kg/m^2^ (4.8) | Body Fat % | 22.3 (7.5) | 23.4 (8.6) | 3% higher total body lean mass  No difference on body fat % between children with T1D and TDC |
|  |  |  |  |  | Male  18.9 (6.1) | 18.1 (8.0) |  |
|  |  |  |  |  | Female  27.8 (6.4) | 27.7 (6.4) |  |
|  |  |  |  | Lean mass (kg) | 43.0 (5.8) | 41.6 (7.1) |  |
| Moyer-Mileur et al. (2008) US (36) | Cross-sectional | N=11 (11F) Age: 12.9yrs (1.0) Height (SDS): 0.1 (1.0) BMI: 20.9kg/m^2^ (1.9) Disease duration: 5.9yrs (3.7) HbA1c: 8.1% (1.0)  Insulin dosage: 1.0 U/kg/day (0.2) | N=10 (10F) Age: 13.1yrs (1.1) Height (SDS): 0.2 (0.6) BMI: 20.3kg/m^2^ (2.1) HbA1c: 4.9% (0.3) | Body Fat %  Lean mass (kg) | 29.8 (6.3)  33.4 (5.4) | 29.1 (7.0)  33.5 (5.4) | No difference on body fat % and total body lean mass between children with T1D and TDC |
|  |  |  |  |  |  |  |  |
| Nadeau et al. (2010) *US* (37) | Cross-sectional | N=12 (6M,6F) Age: 14.8yrs (2.6) BMI: 20.9 kg/m² (3.1) Disease duration: 7.5yrs (4)  HbA1c: 8.7% (1.6) | N=12 (6M,6F) Age: 15.6yrs (1.8) BMI: 21.0 kg/m² (2.4) HbA1c: 4.9% (0.3) | Body fat % | 22.0 (6.6) | 20.4 (10.6) | No difference on body fat % and lean mass %, total body fat and lean mass between children with T1D and TDC |
|  |  |  |  | Fat mass (kg) | 13.4 (5.9) | 11.01 (6.4) |  |
|  |  |  |  | Lean mass % | 77.3 (6.6) | 79.4 (10.6) |  |
|  |  |  |  | Lean mass (kg) | 42.4 (6.5) | 44.8 (8.4) |  |
| Parthasarathy et al. (2016) India (38) | Follow-up | N=80 (39M, 41F) Age: 10.7yrs (3.4) Height: 132.3cm (18.1) Height (Z-score): –0.9 (1.1) Body mass: 28.9kg (11.8) Body mass (Z-score): –1 (0.9) BMI: 15.6kg/m^2^ (3.1) BMI (Z-score): –0.7 (0.8) HbA1c: 10% (2) | N=54 Age: 11.7yrs (2.8) Height: 144.7cm (15.2) Height (Z-score): 0 (0.9) Body mass: 35.0kg (11.2) Body mass (Z-score): –0.8 (3.4) BMI: 17.1kg/m^2^ (5.8) BMI (Z-score): –1.3 (5.7) | Body fat % | 20.3 (9.1) | 26.5 (12) | 23% less body fat % in children with T1D  No difference on lean mass % |
|  |  |  |  | Lean mass % | 73.9 (9.0) | 70.7 (9.0) |  |
| Saki et al. (2017) Iran (39) | Cross-sectional | N=87 (39M,48F) Age: 12.4yrs (4.2) Height: 146.2cm (20.1) Body mass: 39.7kg (15.3) BMI: 17.8kg/m^2^ (3.2) Disease duration: 4.4yrs (2.8) HbA1c: 10.2% (2.2)  Insulin dose: 0.7 U/kg/day (0.3) | N=87 (39M,48F) Age: 12.4yrs (4.2) Height: 151.1cm (16.4) Body mass: 41.3kg (13.8) BMI: 17.5kg/m^2^ (3) | Body fat % | 28.4 (5.8) | 23.4 (8.1) | 21% higher body fat % and 14% lower total body lean mass in children with T1D  No difference on total body fat mass between children with T1D and TDC |
|  |  |  |  | Fat mass (kg) | 11.0 (5.1) | 9.9 (5.1) |  |
|  |  |  |  | Lean mass (kg) | 26.3 (10.3) | 30.5 (10.3) |  |
| Santiprabhob et al. (2021)  Thailand (40) | Cross-sectional | N=100 (44M,56F)  Age: 14.5yr (2.7)  Height: 156.3cm (11.2)  Height (Z-score): 0.17 (1.2)  Body mass: 52.7kg (14.6)  Body mass (Z-score): 1.1 (1.7)  BMI: 21.2kg/m^2^ (4.0)  BMI (Z-score): 0.4 (1.1)  Disease duration: median 5.8yre [IQR 3.0-9.1]  HbA1c: 8.9% (1.8)  Insulin dosage: 1.2 U/kg/day (0.3) | N=100 (44M,56F)  Age: 14.3yr (2.7)  Height: 158.4cm (11.7)  Height (Z-score): 0.7 (1.2)  Body mass: 54.1kg (15.0)  Body mass (Z-score): 1.4 (1.8)  BMI: 21.2kg/m^2^ (4.1)  BMI (Z-score): 0.4 (1.1)  HbA1c: 5.2% (0.3) | Lean mass (kg) | 34.5 (9.2) | 35.0 (9.4) | No difference on total body lean mass between children with T1D and TDC |
| Sarnblad et al. (2006) Sweden (41) | Follow-up (baseline) | N=23 (23F) Age: 15.7yrs (2.1) Height: 163.8cm (7.9) Body mass: median 65.3kg [IQR 16.0] BMI: median 23.6kg/m^2^ [IQR 2.6] BMI (SDS): 0.8 (0.8) Disease duration: median 6.0yrs [IQR 7.7] HbA1c: 7.6% (1.4)  Insulin dosage: 1.1 U/kg/day (0.3) | N=19 (19F) Age: 15.6yrs (1.9) Height: 164.3cm (6.9) Body mass: median 57.2kg [IQR 24.1] BMI: median 20.5kg/m^2^ [IQR 8.0] BMI (SDS): 0.7 (1.2) | Body fat % | 32.6 (8.3) | 31.0 (9.9) | No difference on body fat %, total body fat and lean mass between children with T1D and TDC |
|  |  |  |  | Fat mass (kg) | 20.7 (7.6) | 20.0 (10.0) |  |
| Sarnblad et al. (2016) Sweden (42) | Cross-sectional | N=44 (44F) Age: 16.4yrs (1.9) Height: 165cm (7) Body mass: 66.7kg (11.0) BMI: 24.5kg/m^2^ (3.3) HbA1c: 70.1mmol/mol (13.2)  Insulin dose: 1.1 U/kg/day (0.3) | N=49 (49F) Age: 16.8yrs (1.7) Height: 166cm (6) Body mass: 64.3kg (11.9) BMI: 23.1kg (3.7) | Body fat % | 34.9 (7.6) | 32.2 (8.3) | No difference on body fat % between children with T1D and TDC |
| Vinovskis et al. (2020) US (43) | Cross-sectional | N=50 (25M,25F) Age: 16.0yrs (3.0) Body mass: 67.5kg (17.6) BMI: 23.4kg/m^2^ (5.1) Disease duration: 5.7yrs (2.6) HbA1c: 8.7% (1.3) | N=20 (6M,14F) Age: 16.1yrs (2.9) Body mass: 62.7kg (14.5) BMI: 22.7kg/m^2^ (3.7) HbA1c: 5.2% (0.2) | Fat mass (kg) | 20.3 (9.4) | 19.2 (6.7) | No difference on total body fat mass between children with T1D and TDC |
| Whalley et al. (2009)  New Zealand (44) | Cross-sectional | N=11 (11F)  Age: 15.5yrs (1.1)  Height: 163cm (6)  Body mass: 65.0kg (9.89)  BMI: 24.5kg/m^2^ (3.6)  Disease duration: median 66 months [IQR 13-128]  HbA1c: 8.7% (1.0) | N=9 (9F)  Age: 14.9yrs (1.182)  Height: 167cm (7)  Body mass: 58.1kg (7.9)  BMI: 20.8kg/m^2^ (2.0)  HbA1c: 5.1% (0.21) | Body fat % | 35.9 (7.8) | 28.1 (5.7) | 28% higher body fat % in children with T1D |
| Wu et al. (2021) China (45) | Cross-sectional | N=48 (18M,30F)  Age: 14.0yrs (2.9)  Height: 160cm (13)  Body mass: 49.9kg (12.6)  BMI: 19.0kg/m^2^ (3.1)  BMI (Z-score): -0.3 (1.2)  Disease duration: 3.6yrs (2.3)  HbA1c: 61mmol/mol (9) or 7.7% (2.5)  Insulin dosage: 0.9 U/kg/day (0.3) | N=19 (9M,11F)  Age: 13.6yrs (3.5)  Height: 159cm (13)  Body mass: 52.3kg (15.5)  BMI: 20.4kg/m^2^ (3.3)  BMI (Z-score): 0.5 (1.0) | Body fat % | 29.3 (9.5) | 28.4 (6.6) | No difference on total body fat % between children with T1D and TDC |

Abbreviations: BMI: body mass index; CI: confidence interval; HbA1c: Hemoglobin A1c; IQR: interquartile range; SD: standard deviation; SDS: standard deviation score; SE: standard error; T1D: type 1 diabetes mellitus; TDC: typically developing children; U/kg/day: unit per kilogram per day.

^*^Median, interquartile range, range, standard error, least square mean, and n specified below if mean (SD) not reported and n was specific to the results.

^†^Study characteristics and key findings shown at 1-year follow-up (disease duration = 1 year), since baseline was measured at diagnosis

**Supplementary Table 5.** Risk of bias assessment based on quality assessment from modified Newcastle-Ottawa Scale (17). Good and fair study quality translate to low and moderate risk of bias, respectively

|  | Selection  (0-5 stars)* | Comparability  (0-2 stars) | Outcomes  (0-3 stars) | Overall  (0-9 stars)* | Quality |
| --- | --- | --- | --- | --- | --- |
| Abd El Dayem et al. (2011) (22) | *** | ** | *** | 8 | Good |
| Abd El Dayem & Battah (2012) (23) | *** |  | ** | 5 | Fair |
| Ansell et al. (2020) (24) | *** |  | *** | 6 | Fair |
| Davis et al. (2012) (25) | *** | ** | *** | 8 | Good |
| Devaraja et al. (2020) (26) | *** | ** | *** | 8 | Good |
| Gusso et al. (2017) (27) | *** | ** | *** | 8 | Good |
| Heap et al. (2004) (28) | *** | ** | *** | 8 | Good |
| Ingberg et al. (2003) (29) | *** |  | ** | 5 | Fair |
| Joseph et al. (2020) (30) | *** | ** | *** | 8 | Good |
| Karaguzel et al. (2006) (31) | *** | ** | *** | 8 | Good |
| Komatsu et al. (2005) (32) | *** | ** | *** | 8 | Good |
| Krishnan et al. (2011) (33) | *** | * | *** | 7 | Good |
| Maggio et al. (2010) (34) | *** | ** | *** | 8 | Good |
| Moyer-Mileur et al. (2004) (35) | *** | ** | *** | 8 | Good |
| Moyer-Mileur et al. (2008) (36) | *** | ** | *** | 8 | Good |
| Nadeau et al. (2010) (37) | *** | ** | *** | 8 | Good |
| Parthasarathy et al. (2016) (38) | *** |  | ** | 5 | Fair |
| Saki et al. (2017) (39) | *** | ** | *** | 8 | Good |
| Santiprabhob et al. (2021) (40) | **** | * | ** | 8 | Good |
| Sarnblad et al. (2006) (41) | *** |  | *** | 6 | Fair |
| Sarnblad et al. (2016) (42) | *** | ** | *** | 8 | Good |
| Vinovskis et al. (2020) (43) | *** | ** | *** | 8 | Good |
| Whalley et al. (2009) (44) | *** | ** | *** | 8 | Good |
| Wu et al. (2021) (45) | *** | * | *** | 7 | Good |

*The highest possible score is 9, since question 3 in “Selection” section (non-respondents) was not applicable for all studies.

**Supplementary Table 6.** Meta-regression results correlating potential explanatory factors to the difference in means of body fat %

|  | **Number of Studies** | **Unstandardized** $\boldsymbol{\beta}$ | **95% Confidence Interval** | ***p*-value** |
| --- | --- | --- | --- | --- |
| Sex (female ratio) | 18 | 4.7 | -3.7, 13.2 | 0.272 |
| Age | 17 | 0.3 | -1.0, 1.6 | 0.656 |
| Height | 13 | 0.1 | -0.2, 0.3 | 0.276 |
| Body Mass | 12 | 0.1 | -0.1, 0.3 | 0.277 |
| BMI | 16 | 0.5 | -0.6, 1.5 | 0.399 |
| HbA1c | 18 | -0.5 | -3.8, 2.9 | 0.784 |
| Age of onset | 12 | -2.3 | -3.5, -1.0 | <0.001 |
| Disease duration | 13 | 1.3 | -0.2, 2.7 | 0.085 |
| Insulin dose | 13 | 18.1 | 3.5, 32.6 | 0.015 |

**(A)** Lean mass (kg)
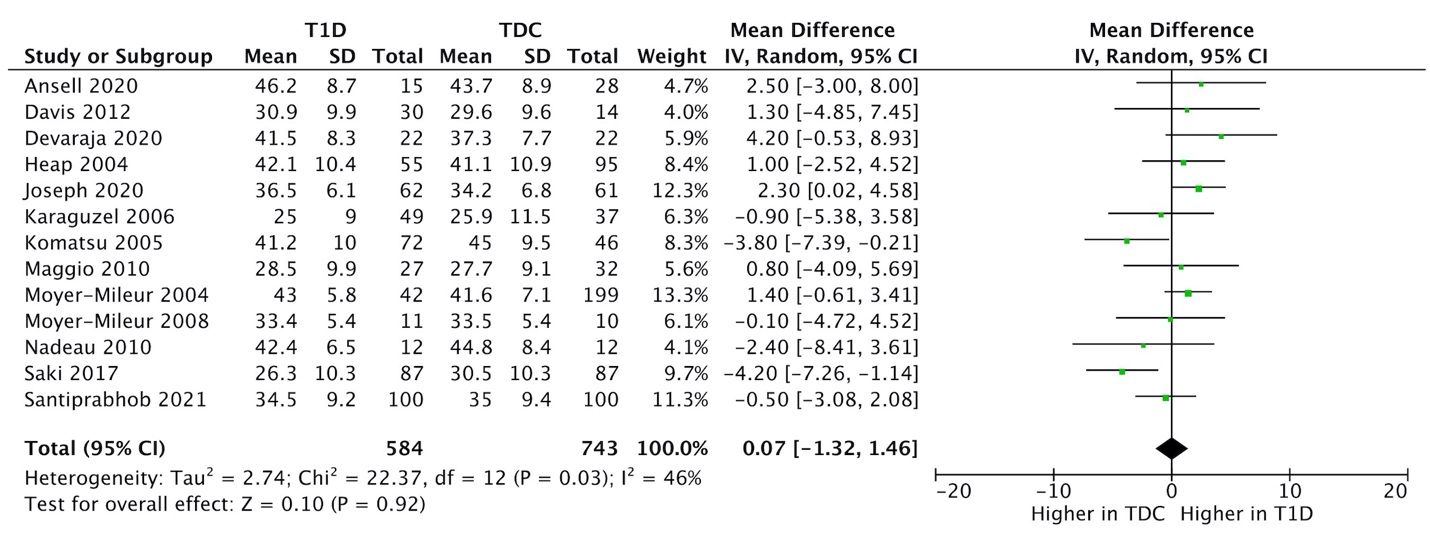


**(B)** Lean mass %

**
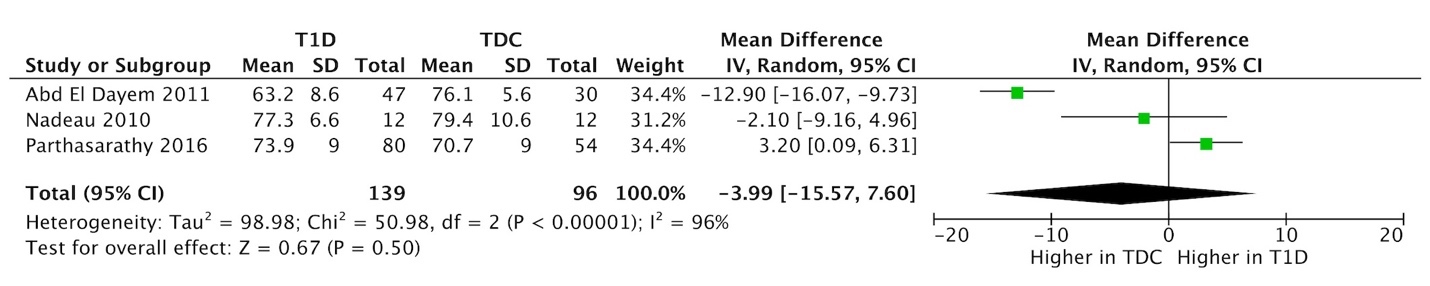
**

**Supplementary Figure 1.** Forest plots of total body **(A)** lean mass (kg), and **(B)** lean mass (%)

**(A)** Fat mass (kg)
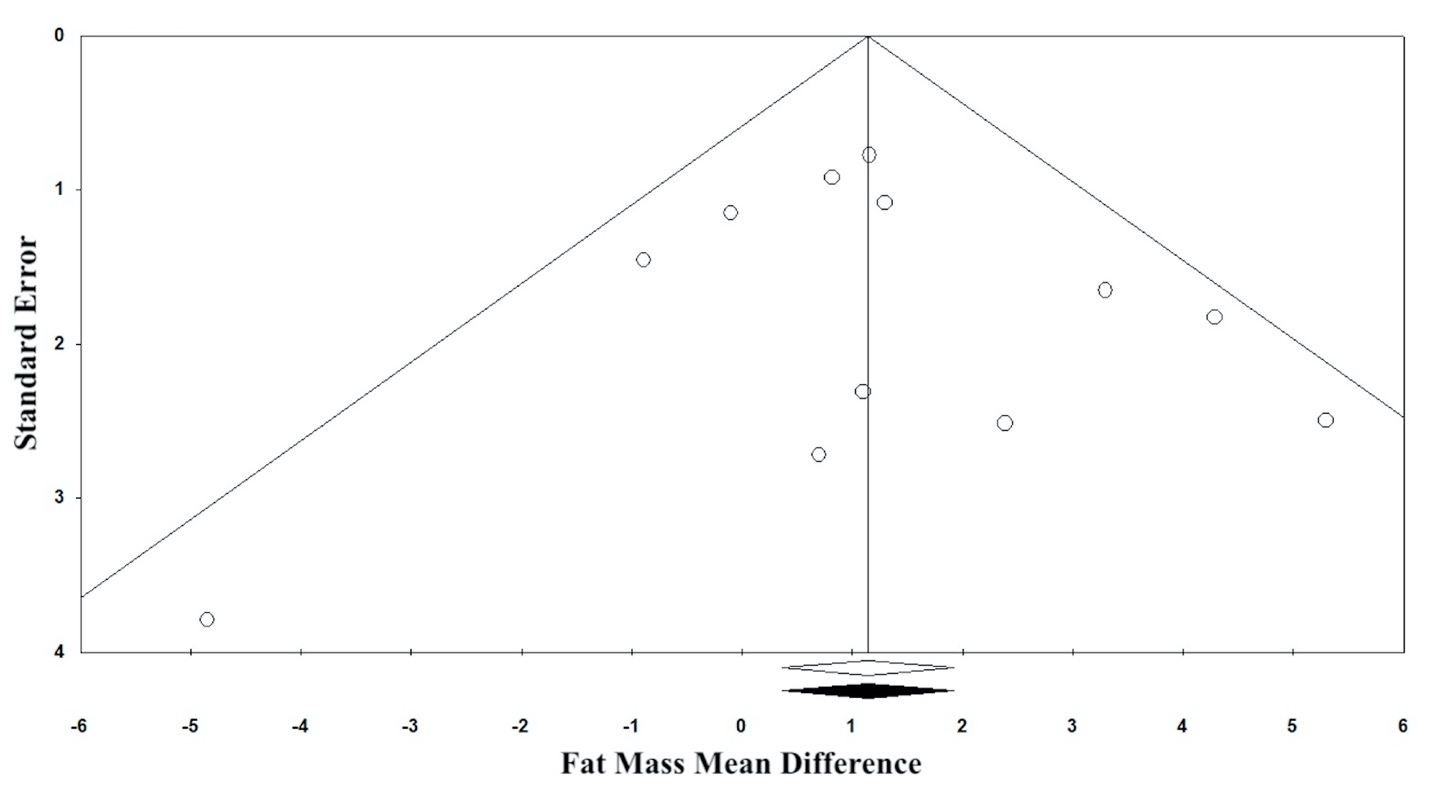


**(B)** Body fat %


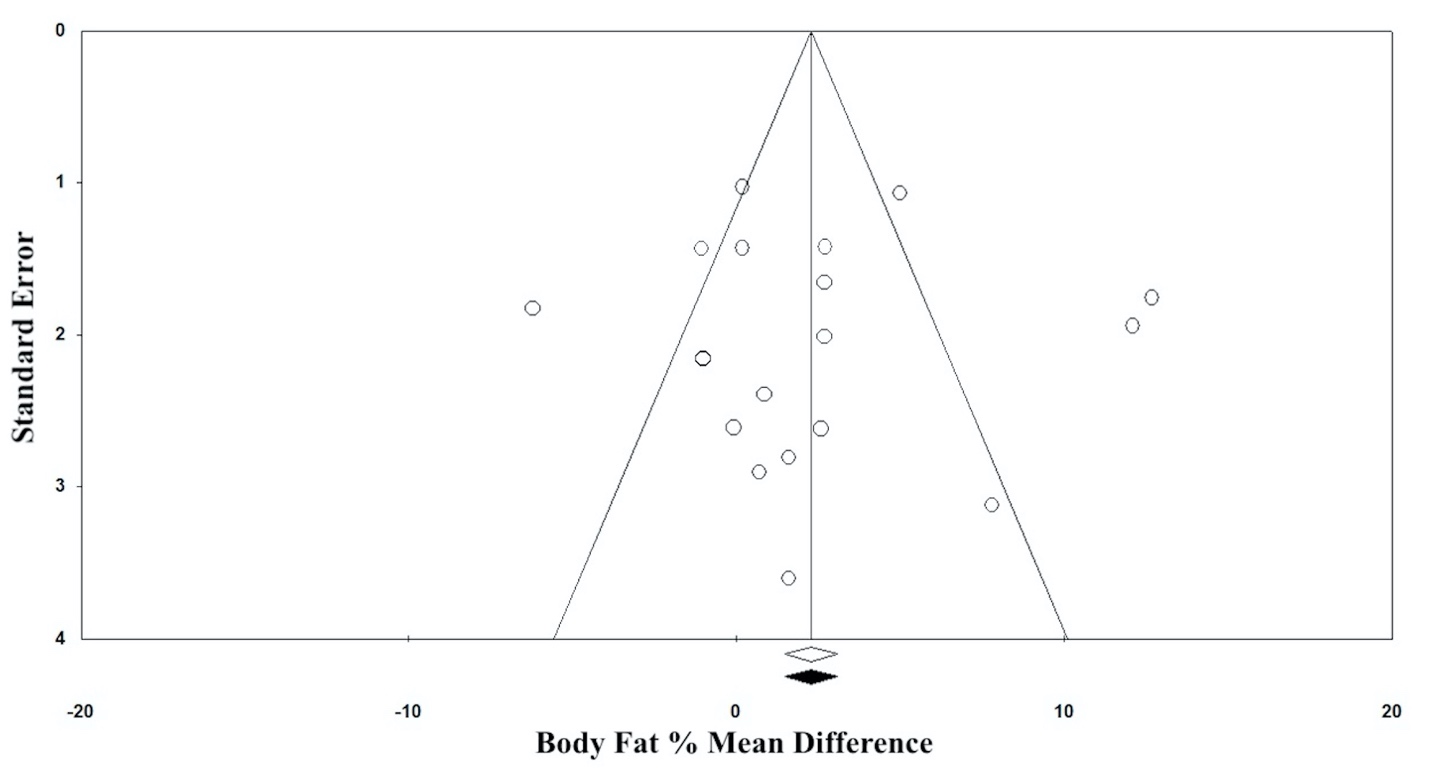


**(C)** Lean mass (kg)
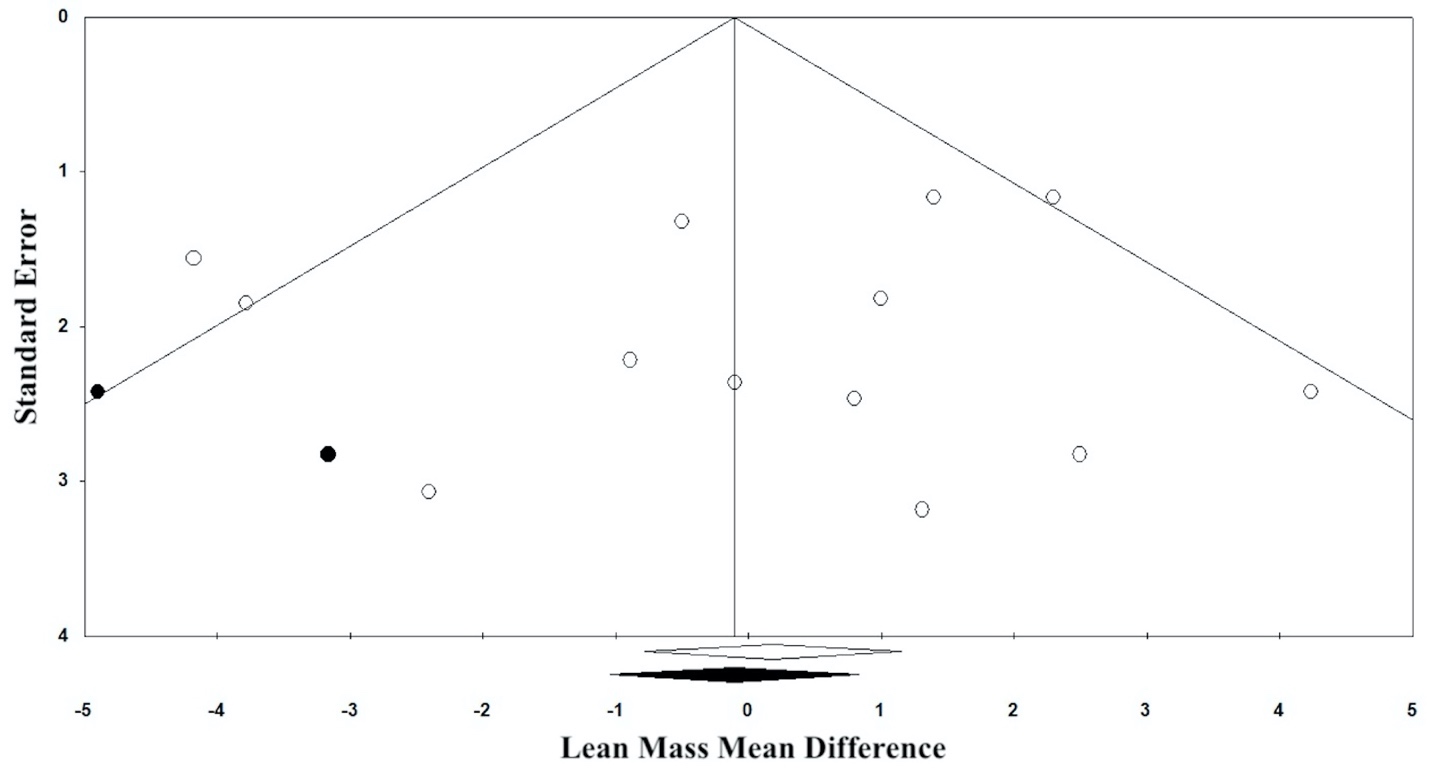


**(D)** Lean mass (%)


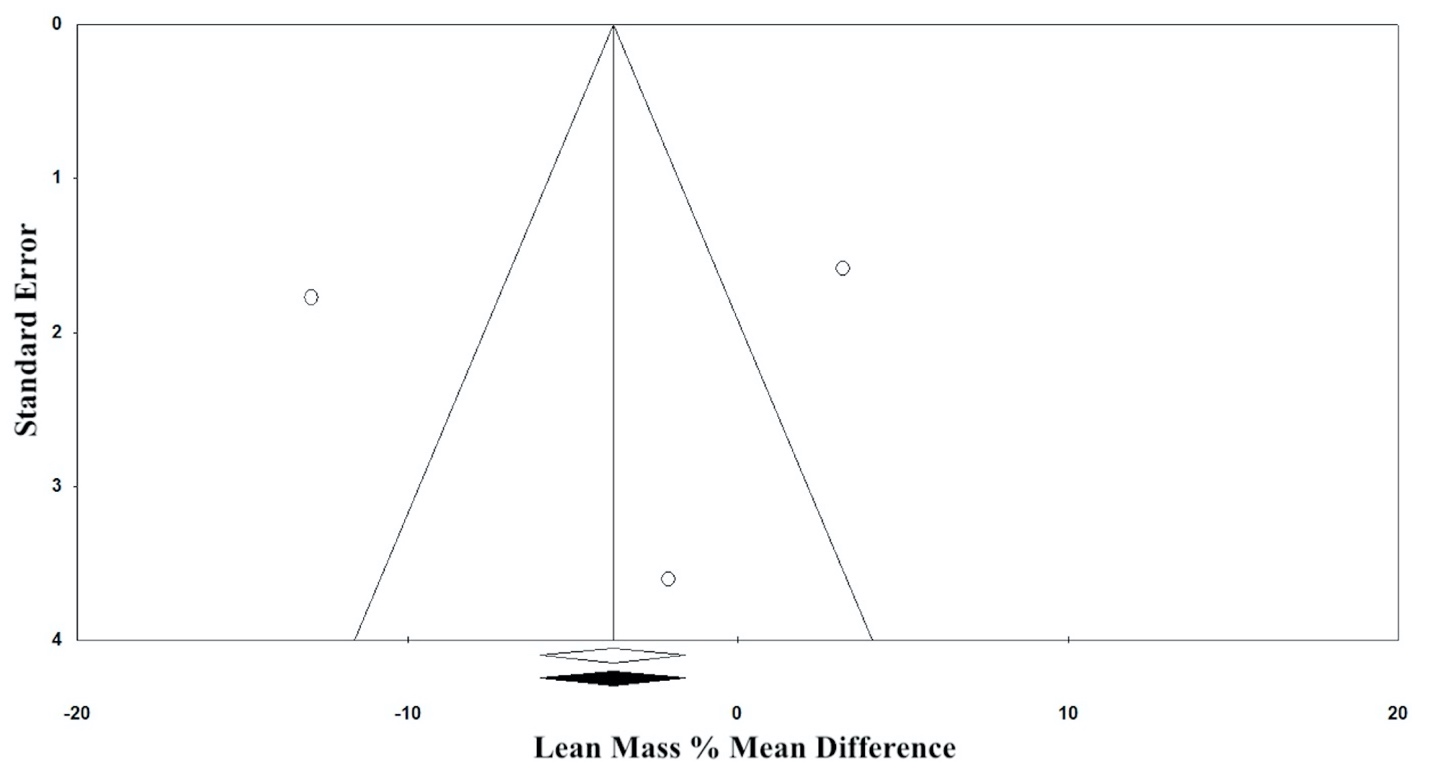


**Supplementary Figure 2.** Re-displayed funnel plots for **(A)** fat mass (kg), **(B)** body fat %, **(C)** lean mass (kg), and **(D)** lean mass % after Duval and Tweedie’s Trim and Fill adjustment

**Reference**

1. Wierzbicka E, Swiercz A, Pludowski P, Jaworski M, Szalecki M. Skeletal status, body composition, and glycaemic control in adolescents with type 1 diabetes mellitus. *J Diabetes Res* (2018) **2018**: doi:10.1155/2018/8121634

2. Mosso C, Halabi V, Ortiz T, Hodgson MI. Dietary intake, body composition, and physical activity among young patients with type 1 diabetes mellitus. *J Pediatr Endocrinol Metab* (2015) **28**:895–902. doi:10.1515/JPEM-2014-0334/MACHINEREADABLECITATION/RIS

3. Castro-Correia C, Santos-Silva R, Pinheiro M, Costa C, Fontoura M. Metabolic risk factors in adolescent girls with type 1 diabetes. *J Pediatr Endocrinol Metab* (2018) **31**:631–635. doi:10.1515/JPEM-2018-0053

4. Jensen RB, Bytoft B, Lohse Z, Johnsen SK, Nielsen MF, Oturai PS, Højlund K, Damm P, Clausen TD, Jensen DM. Impact of Lean Body Mass and Insulin Sensitivity on the IGF-1-Bone Mass Axis in Adolescence: the EPICOM Study. *J Clin Endocrinol Metab* (2021) **106**:E772–E781. doi:10.1210/CLINEM/DGAA861

5. Verroken C, Pieters W, Beddeleem L, Goemaere S, Zmierczak H-G, Shadid S, Kaufman J-M, Lapauw B. Cortical Bone Size Deficit in Adult Patients With Type 1 Diabetes Mellitus. *J Clin Endocrinol Metab* (2017) **102**:2887–2895. doi:10.1210/JC.2017-00620

6. Diamond F, Eichler D, Mayes D, Jorgensen V, Duckett G, Hu C, Cuthbertson D, Root A. Leptin binding activity (LBA) in plasma of nondiabetic and diabetic adolescents and obese children: relation to auxologic and hormonal data. *J Pediatr Endocrinol Metab* (2000) **13**:141–148. doi:10.1515/JPEM.2000.13.2.141

7. Ahmed ML, Ong KKL, Watts AP, Morrell DJ, Preece MA, Dunger DB. Elevated leptin levels are associated with excess gains in fat mass in girls, but not boys, with type 1 diabetes: Longitudinal study during adolescence. *J Clin Endocrinol Metab* (2001) **86**:1188–1193. doi:10.1210/jcem.86.3.7320

8. Bjornstad P, Schäfer M, Truong U, Cree-Green M, Pyle L, Baumgartner A, Reyes YG, Maniatis A, Nayak S, Wadwa RP, et al. Metformin improves insulin sensitivity and vascular health in youth with type 1 diabetes mellitus: Randomized controlled trial. *Circulation* (2018) **138**:2895–2907. doi:10.1161/CIRCULATIONAHA.118.035525

9. Bjornstad P, Cree-Green M, Baumgartner A, Maahs DM, Cherney DZ, Pyle L, Regensteiner JG, Reusch JE, Nadeau KJ. Renal function is associated with peak exercise capacity in adolescents with type 1 diabetes. *Diabetes Care* (2015) **38**:126–131. doi:10.2337/dc14-1742

10. Ismail NA, Abd ElBaky AMNE, Ibrahim MH, Ezzat WM, Elhosary YA, Mostafa EA, Ahmed HH, Rasheed IA. The role of serum cytokeratin 18 and platelet count as non-invasive markers in the diagnosis of nonalcoholic fatty liver disease in children with type 1 diabetes mellitus. *Pediatr Pol* (2020) **95**:141–148. doi:10.5114/polp.2020.100990

11. Krishnan S, Fields DA, Copeland KC, Blackett PR, Anderson MP, Gardner AW. Sex differences in cardiovascular disease risk in adolescents with type 1 diabetes. *Gend Med* (2012) **9**:251–258. doi:10.1016/j.genm.2012.05.003

12. Léger J, Marinovic D, Alberti C, Dorgeret S, Chevenne D, Marchal CL, Tubiana-Rufi N, Sebag G, Czernichow P. Lower bone mineral content in children with type 1 diabetes mellitus is linked to female sex, low insulin-like growth factor type I levels, and high insulin requirement. *J Clin Endocrinol Metab* (2006) **91**:3947–3953. doi:10.1210/jc.2006-0711

13. Roe TF, Mora S, Costin G, Kaufman F, Carlson ME, Gilsanz V. Vertebral bone density in insulin-dependent diabetic children. *Metabolism* (1991) **40**:967–71. doi:10.1016/0026-0495(91)90074-7

14. Roh JG, Yoon JS, Park KJ, Lim JS, Lee HS, Hwang JS. Evaluation of bone mineral status in prepuberal children with newly diagnosed type 1 diabetes. *Ann Pediatr Endocrinol Metab* (2018) **23**:136–140. doi:10.6065/apem.2018.23.3.136

15. Soto N, Pruzzo R, Eyzaguirre F, Iñiguez G, López P, Mohr J, Pérez-Bravo F, Cassorla F, Codner E. Bone mass and sex steroids in postmenarcheal adolescents and adult women with Type 1 diabetes mellitus. *J Diabetes Complications* (2011) **25**:19–24. doi:10.1016/j.jdiacomp.2009.10.002

16. Wierzbicka E, Szalecki M, Pludowski P, Jaworski M, Brzozowska A. Vitamin D status, body composition and glycemic control in Polish adolescents with type 1 diabetes. *Minerva Endocrinol* (2016) **41**:445–455.

17. Modesti PA, Reboldi G, Cappuccio FP, Agyemang C, Remuzzi G, Rapi S, Perruolo E, Parati G. Panethnic differences in blood pressure in Europe: A systematic review and meta-analysis. *PLoS One* (2016) **11**:e0147601. doi:10.1371/journal.pone.0147601

18. Dencker M, Thorsson O, Lindén C, Wollmer P, Andersen LB, Karlsson MK. BMI and objectively measured body fat and body fat distribution in prepubertal children. *Clin Physiol Funct Imaging* (2007) **27**:12–16. doi:10.1111/j.1475-097X.2007.00709.x

19. Hudda MT, Fewtrell MS, Haroun D, Lum S, Williams JE, Wells JCK, Riley RD, Owen CG, Cook DG, Rudnicka AR, et al. Development and validation of a prediction model for fat mass in children and adolescents: Meta-analysis using individual participant data. *BMJ* (2019) **366**: doi:10.1136/bmj.l4293

20. McCarthy HD, Cole TJ, Fry T, Jebb SA, Prentice AM. Body fat reference curves for children. *Int J Obes* (2006) **30**:598–602. doi:10.1038/sj.ijo.0803232

21. Freedman DS, Wang J, Maynard LM, Thornton JC, Mei Z, Pierson RN, Dietz WH, Horlick M. Relation of BMI to fat and fat-free mass among children and adolescents. *Int J Obes* (2005) **29**:1–8. doi:10.1038/sj.ijo.0802735

22. Abd El Dayem SM, El-Shehaby AM, Abd El Gafar A, Fawzy A, Salama H. Bone density, body composition, and markers of bone remodeling in type 1 diabetic patients. *Scand J Clin Lab Invest* (2011) **71**:387–393. doi:10.3109/00365513.2011.573574

23. Abd El Dayem SM, Battah AA. Hypertension in type 1 diabetic patients-the influence of body composition and body mass index: An observational study. *Anadolu Kardiyol Derg* (2012) **12**:60–64. doi:10.5152/akd.2012.014

24. Ansell SKD, Jester M, Tryggestad JB, Short KR. A pilot study of the effects of a high-intensity aerobic exercise session on heart rate variability and arterial compliance in adolescents with or without type 1 diabetes. *Pediatr Diabetes* (2020) **21**:486–495. doi:10.1111/PEDI.12983

25. Davis NL, Bursell JDH, Evans WD, Warner JT, Gregory JW. Body composition in children with type 1 diabetes in the first year after diagnosis: Relationship to glycaemic control and cardiovascular risk. *Arch Dis Child* (2012) **97**:312–315. doi:10.1136/archdischild-2011-300626

26. Devaraja J, Jacques R, Paggiosi M, Clark C, Dimitri P. Impact of Type 1 Diabetes Mellitus on Skeletal Integrity and Strength in Adolescents as Assessed by HRpQCT. *JBMR Plus* (2020) **4**: doi:10.1002/jbm4.10422

27. Gusso S, Pinto T, Baldi JC, Derraik JGB, Cutfield WS, Hornung T, Hofman PL. Exercise training improves but does not normalize left ventricular systolic and diastolic function in adolescents with type 1 diabetes. *Diabetes Care* (2017) **40**:1264–1272. doi:10.2337/dc16-2347

28. Heap J, Murray MA, Miller SC, Jalili T, Moyer-Mileur LJ. Alterations in bone characteristics associated with glycemic control in adolescents with type 1 diabetes mellitus. *J Pediatr* (2004) **144**:56–62. doi:10.1016/j.jpeds.2003.10.066

29. Ingberg CM, Särnblad S, Palmér M, Schvarcz E, Berne C, Åman J. Body composition in adolescent girls with Type 1 diabetes. *Diabet Med* (2003) **20**:1005–1011. doi:10.1046/j.1464-5491.2003.01055.x

30. Joseph T V., Caksa S, Misra M, Mitchell DM. Hip structural analysis reveals impaired hip geometry in girls with type 1 diabetes. *J Clin Endocrinol Metab* (2020) **105**:1–9. doi:10.1210/clinem/dgaa647

31. Karagüzel G, Ozdem S, Boz A, Bircan I, Akçurin S. Leptin levels and body composition in children and adolescents with type 1 diabetes. *Clin Biochem* (2006) **39**:788–793. doi:10.1016/j.clinbiochem.2006.02.014

32. Komatsu WR, Lima Gabbay MA, Castro ML, Saraiva GL, Chacra AR, Leite de Barros Neto T, Dib SA. Aerobic exercise capacity in normal adolescents and those with type 1 diabetes mellitus. *Pediatr Diabetes* (2005) **6**:145–149. doi:10.1111/j.1399-543X.2005.00120.x

33. Krishnan S, Copeland KC, Bright BC, Gardner AW, Blackett PR, Fields DA. Impact of Type 1 Diabetes and Body Weight Status on Cardiovascular Risk Factors in Adolescent Children. *J Clin Hypertens* (2011) **13**:351–356. doi:10.1111/j.1751-7176.2010.00395.x

34. Maggio ABR, Ferrari S, Kraenzlin M, Marchand LM, Schwitzgebel V, Beghetti M, Rizzoli R, Farpour-Lambert NJ. Decreased bone turnover in children and adolescents with well controlled type 1 diabetes. *J Pediatr Endocrinol Metab* (2010) **23**:697–707. doi:10.1515/JPEM.2010.23.7.697

35. Moyer-Mileur L, Dixon S, Quick J, Askew W, Murray M. Bone mineral acquisition in adolescents with type 1 diabetes. *J Pediatr* (2004) **145**:662–9.

36. Moyer-Mileur LJ, Slater H, Jordan KC, Murray MA. IGF-1 and IGF-binding proteins and bone mass, geometry, and strength: Relation to metabolic control in adolescent girls with type 1 diabetes. *J Bone Miner Res* (2008) **23**:1884–1891. doi:10.1359/jbmr.080713

37. Nadeau KJ, Regensteiner JG, Bauer TA, Brown MS, Dorosz JL, Hull A, Zeitler P, Draznin B, Reusch JEB. Insulin resistance in adolescents with type 1 diabetes and its relationship to cardiovascular function. *J Clin Endocrinol Metab* (2010) **95**:513–521. doi:10.1210/jc.2009-1756

38. Parthasarathy L, Chiplonkar S, Khadilkar V, Khadilkar A. Association between metabolic control and lipid parameters in Indian children with type 1 diabetes. *Indian Pediatr* (2016) **53**:39–41. doi:10.1007/s13312-016-0787-2

39. Saki F, Omrani GR, Dabbaghmanesh MH. Dual X-ray absorptiometry body composition and its associated factors in children and adolescence with type 1 diabetes mellitus in South of Iran, a case-control study. *Int J Diabetes Dev Ctries* (2017) **37**:240–247. doi:10.1007/s13410-016-0479-4

40. Santiprabhob J, Charoentawornpanich P, Khemaprasit K, Manpayak T, Kiattisakthavee P, Pipatsathian A, Wannasilp N, Tangjittipokin W. Effect of gender, diabetes duration, inflammatory cytokines, and vitamin D level on bone mineral density among Thai children and adolescents with type 1 diabetes. *Bone* (2021) **153**:116112. doi:10.1016/j.bone.2021.116112

41. Särnblad S, Ekelund U, Åman J. Dietary fat intake predicts 1-year change in body fat in adolescent girls with type 1 diabetes. *Diabetes Care* (2006) **29**:1227–1230. doi:10.2337/dc05-2318

42. Särnblad S, Magnuson A, Ekelund U, Åman J. Body fat measurement in adolescent girls with type 1 diabetes: a comparison of skinfold equations against dual-energy X-ray absorptiometry. *Acta Paediatr Int J Paediatr* (2016) **105**:1211–1215. doi:10.1111/apa.13366

43. Vinovskis C, Li LP, Prasad P, Tommerdahl K, Pyle L, Nelson RG, Pavkov ME, van Raalte D, Rewers M, Pragnell M, et al. Relative hypoxia and early diabetic kidney disease in type 1 diabetes. *Diabetes* (2020) **69**:2700–2708. doi:10.2337/db20-0457

44. Whalley GA, Gusso S, Hofman P, Cutfield W, Poppe KK, Doughty RN, Baldi JC. Structural and functional cardiac abnormalities in adolescent girls with poorly controlled type 2 diabetes. *Diabetes Care* (2009) **32**:883–888. doi:10.2337/dc08-2005

45. Wu N, Bredin SSD, Jamnik VK, Koehle MS, Guan Y, Shellington EM, Li Y, Li J, Warburton DER. Association between physical activity level and cardiovascular risk factors in adolescents living with type 1 diabetes mellitus: a cross-sectional study. *Cardiovasc Diabetol* (2021) **20**: doi:10.1186/S12933-021-01255-0
